# Disparities in Nutritional Adequacy of Diets between Different Socioeconomic Groups of Finnish Adults

**DOI:** 10.3390/nu14071347

**Published:** 2022-03-23

**Authors:** Liisa M. Valsta, Heli Tapanainen, Teea Kortetmäki, Laura Sares-Jäske, Laura Paalanen, Niina E. Kaartinen, Peppi Haario, Minna Kaljonen

**Affiliations:** 1 Department of Public Health and Welfare, Finnish Institute for Health and Welfare, 00271 Helsinki, Finland; liisa.valsta@thl.fi (L.M.V.); laura.sares-jaske@thl.fi (L.S.-J.); laura.paalanen@thl.fi (L.P.); niina.kaartinen@thl.fi (N.E.K.); peppi.haario@thl.fi (P.H.); 2 Department of Social Sciences and Philosophy, University of Jyväskylä, 40014 Jyväskylä, Finland; teea.kortetmaki@jyu.fi; 3 Environmental Policy Centre, Finnish Environment Institute, 00790 Helsinki, Finland; minna.kaljonen@syke.fi

**Keywords:** dietary intake, socioeconomic differences, urbanisation, 24 h dietary recall, usual intake modelling, dietary guidelines, dietary recommendations, macronutrients, micronutrients, climate-friendly

## Abstract

Information on dietary adequacy is needed to assess food and nutrition security in a modern society, especially in the transition towards climate-friendly food systems. In this study, differences in the nutritional adequacy of diets among Finnish adults were evaluated in population groups of different education, income and urbanisation levels. The study used data from the FinDiet 2017 Survey (*n* = 1655, 18–74 years). Modelled usual intakes of foods and nutrients were evaluated relative to food-based dietary guidelines issued by the National Nutrition Council of Finland (FNNC) and with respect to nutrient adequacy following the Nordic Nutrition Recommendations and FNNC. For about half of the nutrients studied, intakes were found to be adequate. Intakes of protein, fat, saturated fatty acids and salt were estimated to be high. By contrast, inadequate intakes were seen in folate and vitamins A, D, B1, B2 and C in almost all groups studied. Groups with a higher education and income, groups that lived in urban areas and, in particular, women adhered more closely to recommended food consumption and nutrient intakes than others. However, major challenges posed by the Finnish diet are common to all groups studied, and only certain dietary features evaluated in view of nutritional adequacy are associated with socioeconomic differences.

## 1. Introduction

The increasing prevalence of diet-related non-communicable diseases (NCDs) is one important motivation to evaluate dietary adequacy as a baseline or a follow-up measure for health and nutrition policy actions [1,2,3]. Dietary intake data that record dietary habits and food consumption as well as nutrient intakes are needed to develop and evaluate health, nutrition and food policy actions at the national level [4,5] and in international coordinations [6,7,8,9]. Improvements in dietary assessment methods and greater harmonisation of surveillance activities have enhanced the accuracy of dietary assessments and the comparability of results internationally [10,11,12]. At this point, more insight is needed into the dietary differences between population groups that differ in their sociodemographic and socioeconomic status (SES) [13,14,15], in order to mitigate SES-based inequalities of health. This information is also increasingly important to predict the rate and the nutritional consequences of the transition towards more climate-friendly diets, i.e., the expected consumption increase in foods of vegetable origin and decrease in animal-based products [16,17,18]. Ensuring a socially just dietary transition requires attentiveness to existing SES-based inequalities in nutrition [15].

Efforts to harmonise dietary assessment methods and tools in Europe have made progress during the past decade [10,19,20,21]. Today, researchers also have available improved statistical modelling methods that permit the use of short-term individual food consumption data for estimations of usual intake [22,23] and, consequently, the evaluation of the dietary adequacy of whole population groups [24] using recent dietary recommendations and guidelines (e.g., [25,26]).

In Finland, gender differences in the quality of diets have been observed for several decades already. Women’s diets have repeatedly been shown to be healthier than men´s [27,28]. SES-related health inequalities have received intense research interest during the past decades as well, and measures such as the National Action Plan to Reduce Health Inequalities 2008–2011 have been put forward [29]. However, no noticeable reduction in health inequalities was seen during the Action Plan period [30]. Correspondingly, a more recent study showed no narrowing of educational health inequalities in Finland between 2011 and 2017. Instead, according to some indicators, the disparities had widened [31]. SES differences between diets have been investigated for decades, but studies in Finland have been carried out based, for the most part, on information obtained by questionnaires on meal patterns, consumption frequencies of indicator foods, such as fresh vegetables, meat and meat products, butter and oil, or by comparing mean intakes at group level [7,32,33,34,35,36,37]. Although studies on SES-based differences in food consumption and nutrient intake exist, recent data from Finland are scarce as most quantitative studies only include data until 2007.

Results so far have shown generally higher vegetable and fruit consumption among higher education and income groups. Educational differences in nutrient intakes during the past decades have been most consistent for vitamin C intake, with a higher intake among high education groups, but differences have also been seen for other nutrients [28,38,39,40]. Results from other countries are in line with findings from Finland and point to poorer quality diets among individuals belonging to lower SES groups. In studies drawing on data from Europe or other high-income countries, the consumption of vegetables and fruit as well as the intake of vitamin C and vitamin D has tended to be lower for lower SES groups, whereas sodium intake has generally been higher for them [14,41,42,43].

Regional differences in food consumption have been less extensively studied in Finland, but the findings suggest more common use of vegetables and fruit in urban areas [44]. In addition, a recent study showed more common use of red and processed meat in rural areas [45]. Similar findings have been observed in other countries as well [46,47].

Evaluations of dietary adequacy based on usual intake and on an average requirement (AR) of micronutrients [25,48] are new in Europe [49] and have not been carried out for different SES groups in Finland before.

The present study aims to evaluate the nutritional adequacy of adult diets in different sociodemographic and socioeconomic population groups (education, income, urbanisation level), using as its baseline usual intake modelling [22] or, where this is not applicable, comparing mean intakes to recommended daily intake (RI) values by applying the most recent dietary reference values used in the Nordic Countries [25] and at the national level [26]. In addition, mean differences in food consumption, nutrient intakes and sources of nutrients in adult diets among these same population groups are estimated. To gain updated insights into the disparities between population groups, we used the most recent food consumption data of the FinDiet 2017 Survey [28], which represent the Finnish contribution to the EU Menu initiative of the European Food Safety Authority (EFSA) [50]. Based on previous findings [51,52], our hypothesis was that the proportion of individuals whose diet complies with current nutrient recommendations is highest among the highest educational and income groups.

## 2. Materials and Methods

### 2.1. Study Population and Data Collection

The FinDiet 2017 Survey data were collected as a sub-sample of and in collaboration with the FinHealth 2017 Study. The study population and data collection methods have been reported in detail before [50,53]. In brief, the FinHealth 2017 Study, a national health examination survey, was carried out in 50 study locations in mainland Finland between January and May 2017. The sampling design of the survey was based on the Health 2000 Survey [54]. For the FinHealth 2017 Study, a representative sample of adults aged 18 years and above (*n* = 10,247) was drawn from the Population Register using one- and two-stage stratified, random sampling. A 30% random sub-sample (*n* = 3099) among those aged 18–74 years of the FinHealth 2017 Study sample was invited to participate in the FinDiet 2017 Survey [28]. Their diet was assessed by two non-consecutive 24-h dietary recalls and recorded by dietary interviewers using the in-house dietary software Finessi (version 5.0.5, Finnish Institute for Health and Welfare, Helsinki, Finland) [55]. The software included the food list and descriptors of the national food composition database Fineli^®^ [56]. Participants were first interviewed during the health examination part of the survey; the second 24-h dietary recall took place by telephone between February and October 2017, with a minimum interval of 8 days between recalls. A picture booklet of food portions was used to estimate portion sizes. The use of food supplements was recorded as well. The final food consumption data consisted of two accepted, non-consecutive 24-h recalls from 1655 participants, i.e., 53% of the original sub-sample. The under-reporting was evaluated following EU Menu methodology [10]; it was found to be on average 21% for the face-to-face interviews and 18% for the telephone interviews.

Background data, e.g., gender and age, were obtained from the sampling frame, information for the SES background variables (education and income) was obtained from the FinHealth 2017 Study questionnaires [57], and information on residential area was obtained from the Population Register Centre (coordinates of the residence of the participants) and Statistics Finland (categorisation of urbanisation level of residential area based on these coordinates). The three educational categories used here—”low”, “middle” and “high” education level—were created by dividing self-reported number of years of fulltime studying (including primary school) in tertiles by sex and birth year. Questions on total household income before tax deductions during the previous year, and on number of adult and underage household members, were utilized to determine income group. The household income question contained ten categories—from “less than EUR 15,000” and “EUR 15,001–25,000” to “more than EUR 90,000” income per year. For this study, the upper limits of the bottom nine original response categories (e.g., EUR 15,000 for the lowest category and EUR 25,000 for the next category), and the lower limit of the highest category (i.e., EUR 90,000) multiplied by two, were divided by weighted sum of household members, assigning a weight of 1.0 to the first adult, 0.7 to additional adults and 0.5 to underage household members [58]. The resulting individual values were grouped into sex-specific quartiles (Qrt), which in turn were combined into three groups: “lowest Qrt”, “middle Qrts” (2.–3. quartiles combined) and “highest Qrt”. Urbanisation levels were categorized as follows: “urban” (urban areas), “semi-urban” (areas near urban areas and rural centres) and “rural” (remote rural areas). The study population and categorisation into these groups is described in Table 1.

### 2.2. Food Consumption and Compatibility with Food-Based Dietary Guidelines

Consumption of foods and dishes was compared between the different SES groups at the ingredient level after disaggregating the consumed foods according to the recipies of the National Food Composition Database, Fineli^®^ [56]. Exceptions to this principle were processed meat products (e.g., sausages) and processed fish products (e.g., canned fish), which were not disaggregated into ingredients but rather quantified as purchased. Food consumption was classified according to the Fineli^®^ food grouping system, of which results are shown for 13 food groups. These food groups include groups that are highlighted in the Finnish food-based dietary guidelines, namely vegetables and fruit, legumes, nuts and seeds, potatoes, red and processed meat (further broken down into beef, pork and sausages), fish and seafood, liquid milk products (including yogurts) and cheese, and cereals. These are also food groups that are expected to change markedly during the dietary transition towards more climate-friendly diets, i.e., the expected consumption increase in foods of vegetable origin and decrease in animal-based products [16,59].

Consumption of four main food groups was compared to the food-based dietary guidelines, which are as follows: vegetables and fruit consumption (500 g/day excluding juices) and red and processed meat intake (no more than 500 g as cooked/week). For milk products, the guideline (which is 5–6 dL of milk and 2–3 slices of cheese) was summed up and expressed as raw milk needed to produce these amounts, taking into account the higher energy intake of men compared to women; this resulted in an approximate guideline value of 900 g/day of raw milk for men and 700 g/day for women. The amount of raw milk was calculated for men as 6 dL (600 g) of liquid milk plus 3 slices, 30 g each, of cheese multiplied by 10 as a commonly used conversion factor from milk to cheese; for women, this was 5 dL (500 g) of liquid milk plus 2 slices, 20 g each, of cheese. The guidelines for cereal products (9 portions and 6 portions for men and women, respectively) were multiplied by an average amount of cereal per portion, i.e., 27 g, for bread and porridge, and other cereal product portions commonly used in Finland, resulting in an approximate rounded guideline of 245 g/day and 160 g/day for men and women, respectively [26].

### 2.3. Nutrient Intake and Evaluation of Adequacy Relative to Reference Values

The mean intakes of 20 macro- and micronutrients from food alone (i.e., excluding food supplements) were analysed and intake differences between the SES groups evaluated. The adequacy evaluation followed a modification of an evaluation protocol previously reported by Steenbergen et al. [49]. The AR of nutrients was used to estimate the proportion of Finnish adults in different SES groups with inadequate intake, using modelled usual intake distributions and the AR cut-point method [48]. If the proportion of a population group reaching the AR level was ≥90% (i.e., the proportion below the AR level was <10%), the nutrient intake was considered “probably adequate”. If <90% of the population group met the AR level, the intake was judged “not adequate”. When the AR was not available, the RI was used as advised by the Nordic Nutrition Recommendations (NNR2012) [25]. According to the NNR2012, if the mean intake of a group is at or above the RI, there is probably a “low prevalence of inadequacy”, and if the mean intake is below the RI, “no firm conclusions can be drawn regarding the prevalence of inadequacy at the group level”. With respect to macronutrients, the RI ranges as % of total energy (E%) were considered. The macronutrient intake of a SES group was considered to be adequate if inside the RI range. Sodium intake was evaluated as salt intake and compared to the Finnish population reference intake of 5 g/day [26]. For iron in pre-menopausal women, RI was used for intake evaluation since one of the underlying assumptions of the AR cut-point method, symmetrical requirement distribution, is not met by this group [48].

Upper limits were evaluated as well. If >2.5% of a population group exceeded the upper limit of the RI range, the intake was judged to be “high”. For micronutrients, the upper intake level (UL) reference values were adopted from the nutrient recommendations of the National Nutrition Council of Finland [26] and were used to estimate the proportion of Finnish adults that may potentially be at risk of adverse effects due to high intake of a certain nutrient. If the proportion of a population group exceeding the UL was larger than 2.5% of the population, the intake was considered “high”; if below, it was considered to be “safe”.

In addition, the usual intake distributions of macronutrients and micronutrients from food only and from food and dietary supplements combined, for both men and women, were evaluated for the whole sample by comparison with the AR values or RI values, as described above.

### 2.4. Statistical Analyses

All analyses were performed for men and women separately. Non-participation bias was corrected using weighting factors, which improves the representativeness of the results with respect to the Finnish adult population overall [60]. The energy under-reporters were identified following the instructions of EU Menu methodology [10].

Mean consumption or intake with 95% confidence intervals (CIs) was calculated using the mean of the data for two days for each subject. Regression analysis was used to test the mean differences between sociodemographic groups. Age was included as a covariate in the regression models. Consumption or intake data were transformed prior to regression analysis using either log or cube root transformation to achieve normality. For pair-wise tests, multiple comparisons were taken into account using the Tukey–Kramer adjustment. For some episodically consumed food groups, it was not possible to transform the consumption data into a normal distribution. For these, the Kruskal–Wallis non-parametric test was used. Pair-wise comparisons were not performed for non-parametric tests. For all food groups, consumption adjusted for energy intake (g/MJ) was used in statistical tests. Usual intake and the proportion of the population below or above the reference value were estimated with statistical program SPADE (R package SPADE.RIVMNwCore 4.0.92, RIVM, Bilthoven, The Netherlands) [22]. The 95% CIs of the proportions were generated by the bootstrap function available in SPADE, with 500 iterations. Significant differences in proportions between sociodemographic groups were evaluated by non-overlapping 95% CIs.

## 3. Results

### 3.1. Population Characteristics

The characteristics of the whole population, including gender, SES groups and urban–rural categorisation, are shown in Table 1. The sample was evenly divided among educational groups, but the proportions of subjects in the middle income and the urban groups were larger compared to other groups within the income and urbanisation sets, respectively.

### 3.2. Food Consumption by Gender, SES Groups and Urbanisation

The consumption of different foods at ingredient level by gender, SES groups and urbanisation level is shown in Table 2. The highest educated group consumed more vegetables and fruit but less red and processed meat compared to the two lower educated groups. The same pattern, i.e., the highest educated group consuming more foods of vegetable origin and fewer of animal origin, could also be seen in the consumption of nuts and seeds and pork (general test statistically significant). Similar findings with respect to education level were seen within both genders. In addition, the lowest educated group of women consumed less cheese compared to the middle education group.

The highest income group consumed more vegetables and fruit, whereas the consumption of cereals was more likely to be lower among men and women in the highest income group when compared to the lower income groups. Among men, potatoes were consumed less in the highest income group compared to the middle income group (Table 2).

Urban men consumed more vegetables and fruit compared to semi-urban or rural men. Rural men consumed more potatoes and liquid milk compared to urban men. Semi-urban men consumed more red and processed meat than urban men. Rural women consumed more potatoes, red and processed meat and milk fats compared to urban women. Semi-urban women did not differ from urban women regarding potato and milk fat consumption, but consumed more red and processed meat and liquid milk than urban women (Table 2).

### 3.3. Food Consumption in Relation to Food-Based Dietary Guidelines

**Figure 1:**
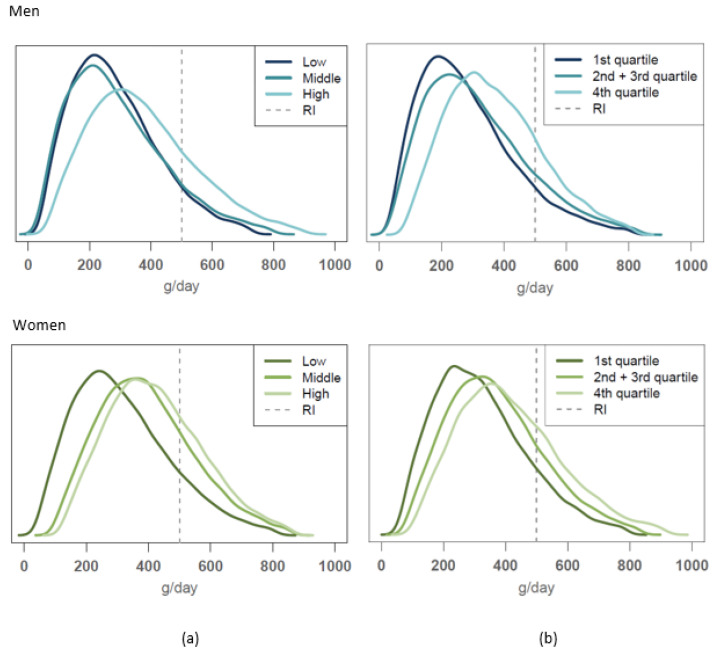
Usual intake distribution of vegetables and fruit consumption compared to the dietary guideline (recommended daily intake (RI); marked as dashed line) of minimally 500 g/day among men (upper figures) and women (lower figures) according to (**a**) educational group, (**b**) income level.

**Figure 2:**
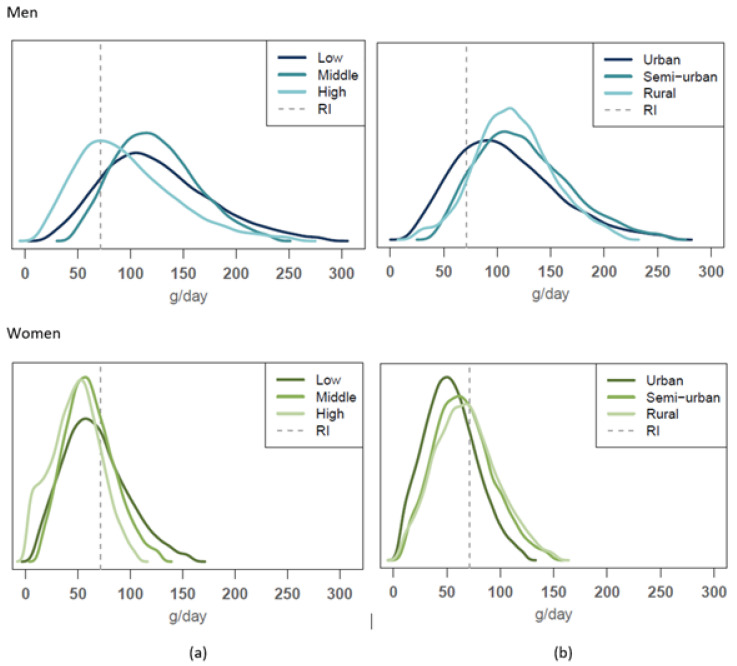
Usual intake distribution of red and processed meat consumption compared to the dietary guideline (RI; marked as dashed line) of a maximum of 500 g/week (= 71 g/day as cooked meat) among men (upper figures) and women (lower figures) according to (**a**) educational group and (**b**) urbanisation level.

The usual food consumption distribution obtained in this study for vegetables and fruit, red and processed meat, milk products and cereals were compared to the reference consumption levels given by the Finnish food-based dietary guidelines. The comparisons for vegetables and fruit and for red and processed meat are shown in Figure 1 and Figure 2, respectively.

The vegetable and fruit consumption guideline (500 g/day) was met by 20–29% of the highest educational and income groups of men and women as well as by 24% of the middle educational group of women (Figure 1). In these SES groups, the proportion of those who reached the guideline was significantly higher compared to other SES groups. On average, 8–15% of men in different urbanisation groups, and 16–23% of women in these groups, met the vegetables and fruit consumption guideline, but differences between the urbanisation groups were not statistically significant within genders.

Overall, 18–22% of men met the guideline value for red and processed meat consumption (no more than 500 g/week as cooked). Among men, the groups adhering more closely to the guideline than others were the highest educated and urban men (34% and 24%, respectively) (Figure 2). There was no significant difference by income level. Among women, adherence to the meat guideline was generally higher; the overall proportion not exceeding the recommended intake was highest among the highest educated of these (83%) and also higher among urban women (77%) compared to other urbanization levels (Figure 2, bottom). Again, income was not a significant factor.

About half the men and women met the milk guideline, but no differences were seen between SES groups. Among men, only 1–6% consumed enough cereals to reach the consumption guideline. No differences were found among men between the SES groups. Among women, the cereal consumption guideline was met best by the lowest income group (15%) and worst among the highest income group (4%). There were no differences between educational or urbanisation levels.

### 3.4. Nutrient Intake Differences and Intake Adequacy Evaluation Based on RI

The evaluation of nutrient intake differences and the adequacy evaluation for nutrients that did not have AR values available and for which the evaluation was based on RI values instead (i.e., macronutrients and salt) are shown in Table 3. In addition, RI was also used for iron in pre-menopausal women. Proportions of population groups meeting the RI reference intakes according to our data on intake distributions in men and women by education, income and urbanisation level are presented in the Supplementary Material, Table S1.

#### 3.4.1. Education

Energy intake varied between 9.4 MJ/day and 9.5 MJ/day among men across educational groups (NS). Energy intake varied between 7.1 MJ/day and 7.9 MJ/day among women across educational groups, being highest for the highest educational group. In men, fat intake was higher in the middle education group than in the lowest education group. The highest educational group had the highest intakes of fibre and total polyunsaturated fatty acid (PUFA). It also had higherintake of omega-3 (n-3) PUFA, compared to the middle education group. Similar differences were seen in women. The intake of fibre was higher in the highest and middle education groups compared to the lowest education group, and the intake of PUFA and n-3 PUFA was highest among those in the highest education group (Table 3).

The mean intakes of total fat, protein, total PUFA and n-3 PUFA met the lower bound of the RI reference values. However, total fat, protein, saturated fatty acid (SFA) and salt intakes were found to be high both in men and women. The higher level of recommended protein intake (20 E%) was exceeded by 18–25% of men and by 4–19% of women; there were no differences between educational levels in this regard (Supplementary Material, Table S2). Mean total carbohydrate and fibre intakes were both below the RI reference values, but based on this fact alone, no firm conclusions can be drawn about the adequacy of intakes either in men or in women (Table 3) [25]. The intake of salt in both men and women exceeded the population goal (5 g/day) in over 95% of men and in about 85–90% of women in different educational groups. The mean iron intake of pre-menopausal women fell below the RI reference value; thus, no firm conclusions can be drawn about the adequacy of their intake [25] (Table 3).

#### 3.4.2. Income

Energy intake by income group ranged between 9.3 and 9.9 MJ/day among men and between 7.2 MJ/day and 7.7 MJ/day among women; differences between income groups within genders were not statistically significant. Intake by income did differ notably, however, for several important nutrients. Thus, with respect to fibre, the lowest income group of men had the lowest intake. For protein, the intake was lower in the middle income group compared to the highest income group of men (Table 3). Intakes of fat, PUFA and n-3 PUFA were highest in the highest income group of women, while intake of carbohydrates was highest in the lowest income group of women (Table 3).

The intakes of total fat, protein, total PUFA and n-3 PUFA met the minimum recommendations in all income groups of men and women. The intakes of total fat and protein were sufficient and indeed exceeded recommended levels in all income groups of men and women. The higher level of recommended protein intake was exceeded by 16–33% of men, with the greatest excess recorded in the highest income group, and by 11–21% of women in the income groups, but without differences between groups (Supplementary Material, Table S2). SFA and salt intakes were found to be high throughout: salt intake in both men and women exceeded the population goal (5 g/day) in over 95% of men and in about 85% of women. The mean iron intake of pre-menopausal women in all income groups was below the RI reference value; thus, no firm conclusions can be drawn about the adequacy of their intake (Table 3).

#### 3.4.3. Urbanisation Level

Energy intake was between 9.4 MJ/day and 9.6 MJ/day among men and between 7.2 MJ/day and 7.4 MJ/day among women based on urbanisation level and did not differ by urbanisation level for either gender. In men, the intakes of PUFA and n-3 PUFA were lower in rural areas compared to urban areas. The protein intake of rural men was also lower in comparison to urban and semi-urban men. In a similar trend, in women, urban residents had higher PUFA intakes compared to semi-urban or rural residents (Table 3).

Intakes of total fat, protein, total PUFA and n-3 PUFA met the recommendations in all urbanisation groups. Practically all men and women met the lower limit of the protein intake recommendation range, i.e., 10 E%. Except for semi-urban women, in all population groups evaluated, far below 10% met the saturated fatty acid recommendation, i.e., <10 E%. Thus, total fat, protein and saturated fatty acid intakes were evaluated to be high in both men and women. The intake of salt exceeded the population goal (5 g/day) in more than 95% of all men and in about 80–85% of women in all urbanisation groups (Supplementary Material, Table S2). The mean iron intake of pre-menopausal women at all urbanisation levels was below the RI reference value, so no firm conclusions can be drawn about intake adequacy (Table 3).

### 3.5. Nutrient Intake Differences and Adequacy Evaluation Based on the AR Cut-Point Method

The nutrient intake differences for micronutrients are shown in Supplementary Material, Table S3. The adequacy evaluations based on the AR cut-point method [48] are shown in Table 4, Table 5 and Table 6.

#### 3.5.1. Education

In men, intakes of vitamin E, folate, vitamin C and iron were higher in the highest educational group compared to the two other groups. In women, intakes of vitamin A, folate and vitamin C were higher in the highest and middle education groups compared to the lowest education group. In addition, the intake of vitamin E was highest in the highest education group (Supplementary Material, Table S3).

The intakes of vitamin E, vitamin B12, calcium, iodine and zinc met the AR reference values in all educational groups of men and women. In addition, both men and post-menopausal women met the iron requirement and women the B2 requirement. Intakes of vitamins A, D, B1 and folate were not adequate (Table 4). In addition, among men, the intake of vitamin B2 was not adequate.

#### 3.5.2. Income

The lowest income group of men had the lowest vitamin C intake. For iron, the intake was lower in the lowest income group compared to the highest, whereas for B12, the intake was lower in the middle income group compared to the highest. In women, the intakes of vitamin D, vitamin E, vitamin B2, folate, vitamin B12 and vitamin C were highest in the highest income group (Supplementary Material, Table S3).

The intakes of vitamin E, vitamin B12, calcium, iodine and zinc met the adequacy criteria in all income groups of men and women. In addition, the adequacy criteria for iron were met in all income groups of men and in post-menopausal women. Vitamins A, D, B1, B2, folate and vitamin C levels were evaluated as not adequate, with the exception of the highest income group of men (vitamin C and D), the highest and middle income groups of women (vitamin B2) and vitamin C in all income groups of women (Table 5).

#### 3.5.3. Urbanisation Level

Only a few differences in nutrient intakes for micronutrients by urbanisation level were seen. Calcium intake was higher among rural men compared to urban men. By contrast, vitamin C intake was higher among urban compared to semi-urban men. In women, urban women had higher folate intakes compared to rural women (Supplementary Material, Table S3).

The intakes of vitamin E, vitamin B12, calcium, iodine and zinc met the adequacy criteria in all urbanisation level groups. In addition, all urbanisation level groups of men and post-menopausal women met the iron recommendations. Under 90% of the population met the AR reference values of vitamin A, D, B1, B2, folate and vitamin C. These intakes are thus considered “not adequate”. The exceptions are the intake of vitamin C among urban women and of vitamin B2 among urban and semi-urban women (Table 6).

#### 3.5.4. Case Vitamin C

**Figure 3:**
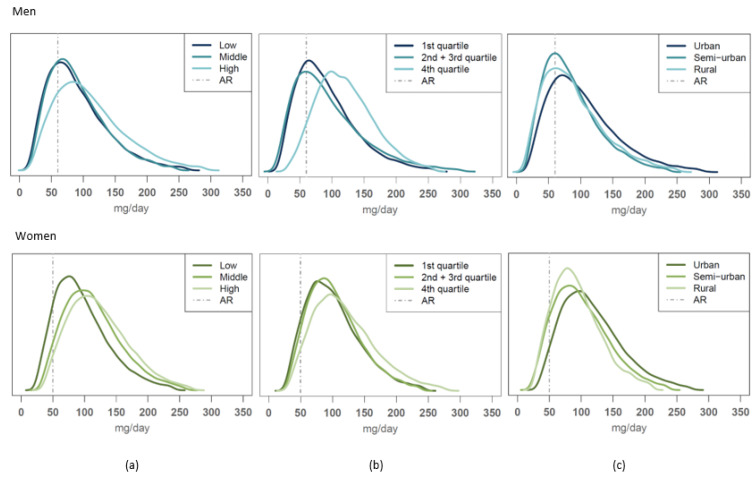
Usual intake distribution of vitamin C intakes compared to the average requirement (AR) value among men (average requirement (AR) = 60 mg/day, upper figures) and women (AR = 50 mg/day, lower figures) according to (**a**) educational group, (**b**) income level and (**c**) urbanization level.

Of all nutrients evaluated, vitamin C intake distributions differed the most between different SES groups. Vitamin C intakes were not adequate for any of the educational groups of men or for the lowest educational group of women. Vitamin C intakes were also not adequate for the two lowest income groups of men or for men at any urbanisation level; semi-urban and rural women had inadequate vitamin C intakes as well (Figure 3). In contrast, in other population groups, over 90% of participants exceeded the AR reference value of vitamin C.

### 3.6. Nutrient Sources of the Different Population Groups Studied

The highest educated group obtained more nutrients from vegetables, fruit and berries, from legumes and nuts, and from fish compared to the lowest educated group, for whom fats, meats, cereals, potatoes and beverages ranked as more important nutrient sources (data only shown for folate in Supplementary Material, Figure S1a–c).

Across income groups, the picture was more mixed. For many nutrients, both the lowest and highest income groups or else two adjacent groups shared the same important food sources; e.g., meat, eggs and legumes served as an important source of folate, and vegetables as an important source of vitamin C, among both the lowest and the highest income groups of men). In this inspection of nutrient sources, meats or legumes were not divided into detailed sub-categories. Among women, the important food groups of the highest income group were more often vegetables, fruit and berries, fish, legumes and nuts, milk, sugars and sweets, whereas with the lower income groups, cereals, meat, potatoes, eggs and fats served more often as important nutrient sources.

Some important food sources of nutrients among urban men and women were fish, vegetables, legumes and nuts, fats, and fruit and berries; by contrast, in semi-urban and rural population groups, meat, eggs, and sugary ingredients and confectionary were seen to be more common. In addition, milk, cereals and potatoes were especially common nutrient sources in the rural population groups.

## 4. Discussion

In this study, the adequacy of the Finnish diet among adults in different SES groups was evaluated to be close to adequate—or the prevalence of inadequacy to be low—in the case of total fat, PUFA, n-3 PUFA, vitamin E, vitamin B12, calcium, iodine and zinc intakes of the whole population and of iron among men and post-menopausal women. On the other hand, improvements are clearly needed to address the levels of high saturated fatty acid and salt intake in all population groups studied. Inadequate intakes were seen for folate, vitamin A, vitamin D, vitamin B1, vitamin B2 as well as vitamin C in almost all SES groups studied. Additionally, protein intake was unnecessarily high, and total carbohydrate and fibre intakes were prone to being below the recommended level across groups.

In the case of most nutrients, either all or none of the studied groups exceeded or did not reach a recommended upper or lower reference intake. This shows that the major challenges in the Finnish diet cover all the groups studied here, and that only a few dietary features evaluated for nutritional adequacy are associated with SES differences. One such exception was the highest income group of men, which had adequate vitamin C and D intakes (>90% of the group reaching the AR value), while the lower income groups did not. Among women, such exceptions were seen in vitamin B2 and C intakes. For vitamin B2, differences in nutritional adequacy were seen by income groups and urbanisation level and for vitamin C by education and urbanisation levels. It was also seen that even when none of the population groups met the threshold for nutritional adequacy set in the evaluation for a certain nutrient, there were differences between the proportions reaching the reference intake. For example, PUFA intakes, which were evaluated to have low prevalence of inadequacy in all studied groups, were nevertheless higher among the highest educational and urban groups compared to the lower educational and rural population groups. Thus, this study shows that attention needs to be paid to nutrition policy actions ensuring the availability of nutritious food for the whole population, but especially for lower SES groups and those living in semi-urban or rural areas. In this study, we covered education, income and urbanisation levels as SES indicators. It may be, though, that age should be a factor of concern as well, insofar as the elderly may need special attention [61].

In general, nutrient intakes in Finland do not differ very much from general European levels [62]. Vitamin D is an exception. In the European context, Finland has one of the highest vitamin D intakes [7]. This is due to the Finnish vitamin D fortification program that was started in the 1940s and was upgraded in 2002 [5,63]. Despite this effort, we estimate vitamin D intake to be adequate only in the highest income group of men (≥90% of the group reaching the AR reference value). However, the differences in the within-group proportion of SES population groups reaching the average vitamin D requirement were shown to be small in our study, insofar as a proportion of over 80% was reached in all groups studied. This is the situation when only food sources of vitamin D are taken into account (excluding food supplement sources). The main sources of vitamin D in the Finnish diet are fortified foods and fish [28,64]. Similarly, there were very few differences between population groups in the proportion of subjects reaching the AR of iodine intake. Iodine has been shown before to be an important nutrient showing lower intakes in lower SES groups in Europe [14]. Again, the small differences in iodine uptake between SES groups in Finland today are due to a salt fortification program in place since the 1940s and upgraded in 2015 [65]. These examples show that food fortification is an effective tool of nutrition policy when it comes to mitigating dietary disparities between different SES or other population groups.

The differences observed in vitamin C intakes here resemble those seen in earlier years in Europe [14]. Vegetables and fruit are the best sources of vitamin C, providing 63% and 71% of vitamin C for men and women, respectively, in the Finnish diet [28]. In this study, men in the highest educational and income groups and urban men and women in the two highest educational groups, women in all income groups and urban women consumed more of these foods compared to other groups within the same gender. Significantly higher proportions in these population groups also met the AR reference value for vitamin C intake. Moreover, although none of the studied groups exceeded the 90% proportion for reaching the AR of folate, a significantly higher proportion of higher-educated men and women and in higher income groups met the AR reference intake for this nutrient. An increase in vegetable and fruit consumption is important, therefore, from the point of view of nutritional equity, health and climate-friendly diets that are rich in foods of vegetable origin.

Protein intake in the Finnish diet stems mainly from animal-based foods (close to 70%) [28]. The fact that red and processed meat intakes among 70–90% of men and among 20–40% of women are above food-based dietary guideline levels in most population groups studied, and that vegetable and fruit consumption is still below guideline levels for about 80% of these groups, provides evidence and motivation for the need to move further towards a more vegetable-based, healthier diet.

We see in this study that women´s food consumption and dietary intakes are closer to dietary guidelines and nutrition recommendations in the case of certain foods (e.g., red and processed meat) and nutrients (e.g., sodium or salt). These are foods and nutrients whose recommended intake represents an upper limit; the same reference criteria (maximum amounts) are given for both genders [25,26]. In this study, the average energy intake of women was 7.3 MJ/day and of men 9.5 MJ/day [28]. This means that staying at or below the maximum recommended intake would require greater dietary adjustments in men, e.g., consumption of less meat/MJ or less salty food compared to women, to ensure similar adequacy outcomes in both genders. In the case of the vegetables and fruit guideline (500 g/day) and certain micronutrients (e.g., vitamin D, folate and calcium) that have the same minimum requirement set for men and women, men have an advantage over women in adequacy evaluation simply because they should and do eat more food. The same applies with respect to different adult age groups, when the same absolute reference values are applied over a broad age range with varying energy needs. In the case of macronutrients (e.g., protein and fatty acids) different energy intakes are taken into account since recommendations are expressed relative to total energy intakes [25,26]. It might be useful in the future to consider whether differences in energy intake between genders should be taken into account more comprehensively in setting dietary guidelines or nutrient recommendations than is done today.

One limitation of this study is that it covered nutrient intake from foods only, excluding food supplements. We address this in the Supplementary Materials, where information on combined intakes of foods and food supplements, and an evaluation of proportions reaching the AR reference values of men and women based on these additional data, are provided (Supplementary Material, Table S4). These data show that some inadequate intakes, e.g., in the case of vitamin D, are resolved by the use of food supplements; but for most nutrients this is not the case.

A known limitation of dietary studies based on self-reporting is the possibility of misreporting, especially an under-reporting of energy consumed. In this study, under-reporting was estimated to be on average about 25–27% for men and about 24–26% for women. These figures are lower than those of national FinDiet Surveys in the past [66]. In certain previous studies, high educational level has shown to predict under-reporting [66], but in other settings, results have been reported to the effect that under-reporting is more prevalent among individuals in lower SES groups [67]. In this study, the reason why higher energy intake among women with high education compared to other educational groups is probably due to differences in reporting of dietary intake. Indeed, women with low or middle educational groups were found to under-report their dietary intake more often than women with high education. This is supported also by the observation of obesity (BMI ≥ 30 kg/m^2^) prevalence being lower among the highest educated women and not the other way around, which is concordant with earlier findings [68]. The fact that our data included approximately 25% of energy under-reporters may have also affected the nutrient adequacy evaluation. It may be possible that the proportion of inadequate diets according to this study is an overestimation of the real situation. This would mean that among women, the lower SES groups would have adequate diets more commonly than was found by this study. If that was the case, this would mean that the disparities between SES groups would be even less among Finnish women than evaluated here. Among men, under-reporting did not differ between the SES groups.

The strengths of this study were the very careful sampling design based on population register data; the data collection methods, involving a selection of supporting tools for data collection; extensive quality controls; standardization interviews for the dietary interviewers throughout the data collection period using the method reported by Gavrieli and co-workers [21]; availability of a broad set of background data substantiating SES variables; data management and weighting methods used to tackle the non-response bias [50,53]. The data collection methods used were in line with the European guidance on methodology for harmonised food consumption data collection in EU member states put forward by EFSA [10]. Advanced modelling methods were used to obtain usual food consumption and nutrient intake estimates and population distributions based on short-term data collection [22], which enabled more accurate dietary adequacy evaluations, using also the AR-based cut-point method [48].

While addressing nutritional disparities and the related health inequalities has been acknowledged as an important societal goal in itself, the increasing pressure to transition to more climate-friendly diets will make this issue even more urgent in the future. Active interventions are needed to achieve these goals in tandem. According to the present study, such interventions should certainly aim for an increase in vegetable and fruit consumption across the entire population; initiatives such as the inclusive school meal program in Finland [69,70,71] still deserve to be fostered. Moreover, the successful fortification programs implemented since the 1940s (Vitamin D, iodine) may become an important way to make sure lower SES classes are quickly brought on board. However, policy makers should also consider more targeted nutritional interventions with respect to meat and dairy consumption. Meanwhile, our results underline the need to update nutritional guidelines while taking into account a more nuanced understanding of SES-based differences. Finally, any initiatives taxing foods on the basis of climate or health impacts should consider their impacts on different SES groups.

## 5. Conclusions

This study shows that the major challenges in the Finnish diet apply to all population groups studied; only certain dietary features affecting nutritional adequacy are associated with SES differences. Urban, higher educated population groups with a higher income –and especially women among these groups—adhere more closely to dietary guidelines and recommended nutrient intakes than other population groups, but not even these groups reach the desired reference intakes of all nutrients evaluated. The dietary transition towards healthier and more climate-friendly diets is more advanced among these trailblazer population groups. Meanwhile, those groups whose protein intake is, for the most part, still based on red and processed meat, i.e., men in general and less educated and non-urban men in particular, will need to make the greatest adjustments.

Means to mitigate nutritional disparities must be applied broadly and pragmatically to safeguard equal opportunities to achieve adequate nutrition and health regardless of a person’s income, education or place of residence. The transition to climate-friendly diets needs to ensure the right to good nutrition for all.

## Figures and Tables

**Table 1:** The participants of the FinDiet 2017 Survey by gender, education, income and urbanisation level.

	Men					Women				
	*n*	%	Mean Age, Years	% Under-Reporters	% BMI ≥30 kg/m^2^	*n*	%	Mean Age, Years	% Under-Reporters	% BMI ≥30 kg/m^2^
Education										
Low	259	33	50.7	26	26	269	31	50.3	29	33
Middle	258	33	50.1	30	23	305	35	51.7	30	26
High	256	33	54.0	24	22	285	33	52.7	15	22
Missing	7	1				16	2			
Income										
Lowest Qrt	200	26	45.4	26	28	187	21	45.5	33	28
Middle (2.–3. Qrt)	389	50	54.8	27	22	419	48	54.7	25	31
Highest Qrt	175	22	51.6	26	22	235	27	50.6	15	18
Missing	16	2				34	4			
Urbanisation level										
Urban	451	58	50.1	29	22	535	61	50.6	24	24
Semi-urban	207	27	53.3	26	26	192	22	52.7	28	28
Rural	122	16	54.9	19	29	147	17	53.8	25	40
Missing	0	0				1	0			
All	780	100	51.7	27	24	875	100	51.6	25	27

BMI, body mass index; Qrt, quartile.

**Table 2:** Average consumption of food groups (g/day) in men and women by education, income and urbanisation level.

	Education								Income								Urbanisation Level							
	Low (1)		Middle (2)		High (3)		General Test	Pair-Wise Comparison	Lowest Qrt (1)		Middle (2.–3. Qrt) (2)		Highest Qrt (3)		General Test	Pair-Wise Comparison	Urban (1)		Semi-Urban (2)		Rural (3)		General Test	Pair-Wise Comparison
Food Group	Mean	95% CI	Mean	95% CI	Mean	95% CI	*p*-Value	Sign. Diff. ^1^	Mean	95% CI	Mean	95% CI	Mean	95% CI	*p*-Value	Sign. Diff. ^1^	Mean	95% CI	Mean	95% CI	Mean	95% CI	*p*-Value	Sign. Diff. ^1^
Men																								
Veg. and fruit	289	265–314	283	257–310	371	340–402	<0.001	1, 2 < 3	280	246–313	313	289–337	360	329–391	<0.001	1, 2 < 3	327	304–349	298	267–329	271	229–312	0.001	1 > 2, 3
Potatoes	89	79–99	86	76–95	79	68–89	NS	NS	84	73–95	91	82–100	71	60–83	0.040	2 > 3	80	72–87	85	77–93	107	91–124	0.021	1 < 3
Legumes	8	5–10	13	7–20	16	10–22	NS	^2^	14	7–21	12	8–15	12	8–16	NS	^2^	15	11–20	8	5–11	7	4–10	NS	^2^
Nuts and seeds	5	3–6	8	5–10	9	6–11	<0.001	^2^	7	4–10	6	5–7	8	5–11	0.001	^2^	8	6–10	6	4–8	4	2–5	0.017	^2^
Red and proc. Meat	146	131–161	149	136–163	117	102–131	<0.001	1, 2 > 3	141	124–158	135	125–145	144	125–163	NS	NS	133	122–144	150	133–167	139	119–160	0.007	1 < 2
Beef	27	21–33	41	32–51	31	23–39	0.008	^2^	37	27–47	28	23–34	39	29–48	NS	^2^	35	29–42	33	26–40	22	17–28	NS	^2^
Pork	42	32–51	42	35–50	24	18–30	<0.001	^2^	43	32–54	37	31–43	28	20–36	NS	^2^	35	29–41	43	32–54	32	24–41	NS	^2^
Sausages	40	32–47	31	26–37	35	25–45	NS	^2^	31	23–40	36	31–42	40	30–49	NS	^2^	31	25–36	41	32–49	46	33–59	0.001	^2^
Fish and seafood	34	27–41	32	16–47	43	35–51	0.032	^2^	34	17–51	35	29–41	39	30–48	0.020	^2^	40	30–49	27	22–32	34	21–48	NS	^2^
Liquid milk	407	358–457	389	329–450	342	301–384	NS	NS	444	385–503	355	320–389	358	301–416	NS	NS	361	320–402	400	339–462	444	376–512	0.016	1 < 3
Milk fats	19	16–22	18	15–21	19	16–22	NS	NS	18	14–22	19	17–21	18	15–21	NS	NS	18	16–20	19	17–22	20	16–23	NS	NS
Cheese	32	28–35	36	30–42	33	29–37	NS	NS	33	27–40	35	31–38	32	27–38	NS	NS	32	28–35	36	30–43	35	29–41	NS	NS
Cereals	146	137–156	154	137–171	150	141–158	NS	NS	157	138–176	152	144–159	139	128–150	0.035	2 > 3	151	140–162	144	133–154	156	144–169	NS	NS
Women																								
Veg. and fruit	326	294–357	394	365–423	422	390–453	<0.001	1 < 2, 3	342	307–377	385	358–412	418	387–448	0.009	1 < 3	393	370–417	375	321–429	340	293–387	NS	NS
Potatoes	64	57–71	64	55–73	58	52–65	NS	NS	61	51–70	65	58–72	58	51–65	NS	NS	59	53–65	60	53–67	76	66–87	0.004	1, 2 < 3
Legumes	11	8–14	14	8–19	15	11–20	0.038	^2^	14	10–19	14	10–19	12	9–16	NS	^2^	15	11–19	11	6–16	9	5–12	NS	^2^
Nuts and seeds	6	4–8	8	6–10	11	9–13	<0.001	^2^	7	5–9	8	6–10	12	9–14	<0.001	^2^	10	8–11	7	5–9	7	4–9	0.005	^2^
Red and proc. meat	79	72–86	72	64–80	61	55–68	<0.001	1, 2 > 3	76	66–85	71	66–77	65	57–73	NS	NS	66	60–71	78	70–86	83	73–94	<0.001	1 < 2, 3
Beef	17	13–22	21	17–26	17	13–21	NS	^2^	18	13–24	18	15–21	20	16–25	NS	^2^	19	16–23	17	14–21	17	14–20	NS	^2^
Pork	20	16–24	19	15–23	14	11–18	0.015	^2^	22	16–28	18	15–22	14	11–17	0.016	^2^	15	13–18	21	16–26	25	17–33	0.006	^2^
Sausages	20	16–25	14	10–17	13	10–16	NS	^2^	19	14–24	16	13–19	11	8–14	NS	^2^	13	11–16	20	13–27	19	14–25	NS	^2^
Fish and seafood	24	19–29	27	22–32	34	28–39	0.011	^2^	22	17–27	27	23–31	35	29–41	0.003	^2^	30	26–34	25	19–30	23	16–30	NS	^2^
Liquid milk	333	295–370	290	260–320	286	260–313	NS	NS	297	257–338	306	282–331	307	274–341	NS	NS	280	258–303	341	303–380	332	278–386	0.043	1 < 2
Milk fats	12	11–14	14	12–15	14	12–15	NS	NS	11	9–14	14	12–15	14	12–16	NS	NS	13	11–14	13	10–15	16	13–19	0.001	1, 2 < 3
Cheese	19	17–21	24	21–26	24	21–27	0.004	1 < 2	20	17–23	23	21–25	24	22–27	NS	NS	22	21–24	22	19–25	22	19–26	NS	NS
Cereals	110	103–117	108	101–116	116	109–122	NS	NS	119	108–129	110	105–115	109	103–115	0.015	1 > 2, 3	110	105–116	114	109–120	109	100–119	NS	NS

^1^ Considered significantly different with group rankings as indicated, if for the general test *p* < 0.05 and for pair-wise comparison *p* < 0.05. ^2^ Pair-wise comparisons not produced for food groups where the non-parametric general test had to be used. CI, confidence interval; Sign. Diff., significant difference; NS, not statistically significant; veg., vegetables; proc., processed.

**Table 3:** Average nutrient intakes and adequacy evaluation based on recommended daily intake (RI) values in men and women by education, income and urbanisation level.

A. Men	Reference Value							General Test	Pair-Wise Comparison	Overall Adequacy Evaluation ^2^
Nutrient	RI	Mean	95% CI	Mean	95% CI	Mean	95% CI	*p*-Value	Sign. Diff. ^1^	
Education		Low (1)		Middle (2)		High (3)				
Energy (MJ)	-	9.4	9.0–9.8	9.5	8.9–10.2	9.5	9.1–9.8	NS	NS	-
Protein (E%)	10–20	17.8	17.4–18.3	18.1	17.6–18.6	18.1	17.5–18.7	NS	NS	High intake
Total Carbohydrates (E%)	45–60	44.2	43.2–45.2	42.6	41.7–43.5	43.2	42.1–44.4	0.036	1 > 2	No firm conclusions can be drawn
Fibre (g)	>35	21.9	20.7–23.2	21.1	20–22.1	24.4	23–25.8	0.001	3 > 1, 2	No firm conclusions can be drawn
Fat (E%)	25–40	38.0	37.1–38.9	39.3	38.5–40.1	38.6	37.6–39.7	0.036	1 < 2	Low prevalence of inadequacy
Saturated f.a. (SFA) (E%)	<10	15.1	14.6–15.5	15.4	14.8–15.9	14.7	14.2–15.2	NS	NS	High intake
Polyunsaturated f.a. (PUFA) (E%)	5–10	6.6	6.4–6.8	6.7	6.5–7	7.1	6.8–7.3	0.023	3 > 1, 2	Low prevalence of inadequacy
N-3 PUFA (E%)	1	1.5	1.5–1.6	1.5	1.4–1.6	1.7	1.6–1.8	0.0163	3 > 2	Low prevalence of inadequacy
Salt (g)	≤5	8.8	8.4–9.1	9.0	8.3–9.7	8.4	8–8.8	NS	NS	High intake
Income		Lowest Qrt (1)		Middle (2)		Highest Qrt (3)				
Energy (MJ)	-	9.9	9.2–10.5	9.3	9.0–9.7	9.4	9.0–9.8	NS	NS	-
Protein (E%)	10–20	18.0	17.4–18.6	17.6	17.2–18	18.8	18.1–19.5	0.041	2 < 3	High intake
Total Carbohydrates (E%)	45–60	43.0	42–44	44.1	43.4–44.9	42.4	41–43.8	NS	NS	No firm conclusions can be drawn
Fibre (g)	>35	21.3	19.8–22.8	23.0	21.9–24.1	23.1	21.6–24.7	0.036	1 < 3	No firm conclusions can be drawn
Fat (E%)	25–40	39.0	38–39.9	38.2	37.5–39	38.8	37.6–40	NS	NS	Low prevalence of inadequacy
Saturated f.a. (SFA) (E%)	<10	15.2	14.6–15.8	15.1	14.8–15.5	14.8	14.2–15.4	NS	NS	High intake
Polyunsaturated f.a. (PUFA) (E%)	5–10	6.8	6.4–7.1	6.7	6.5–6.9	7.0	6.7–7.3	NS	NS	Low prevalence of inadequacy
N-3 PUFA (E%)	1	1.5	1.4–1.6	1.5	1.5–1.6	1.6	1.5–1.8	NS	NS	Low prevalence of inadequacy
Salt (g)	≤5	9.0	8.3–9.7	8.7	8.4–9	8.7	8.3–9.2	NS	NS	High intake
Urbanisation level		Urban (1)		Semi-urban (2)		Rural (3)				
Energy (MJ)	-	9.4	9.0–9.8	9.6	9.1–10.0	9.6	9.1–10.0	NS	NS	-
Protein (E%)	10–20	18.2	17.8–18.6	18.2	17.5–18.9	16.8	16.2–17.3	0.003	1,2 > 3	High intake
Total Carbohydrates (E%)	45–60	43.1	42.2–43.9	43.0	41.9–44.2	45.5	43.5–47.6	NS	NS	No firm conclusions can be drawn
Fibre (g)	>35	22.4	21.4–23.4	22.0	20.5–23.5	23.0	20.9–25	NS	NS	No firm conclusions can be drawn
Fat (E%)	25–40	38.8	37.9–39.6	38.7	37.9–39.5	37.7	35.9–39.5	NS	NS	Low prevalence of inadequacy
Saturated f.a. (SFA) (E%)	<10	14.8	14.4–15.3	15.3	14.8–15.7	15.7	14.8–16.6	NS	NS	High intake
Polyunsaturated f.a. (PUFA) (E%)	5–10	7.0	6.7–7.2	6.7	6.5–6.9	6.2	5.8–6.6	0.006	1 > 3	Low prevalence of inadequacy
N-3 PUFA (E%)	1	1.6	1.5–1.7	1.5	1.4–1.6	1.4	1.3–1.6	0.026	1 > 3	Low prevalence of inadequacy
Salt (g)	≤5	8.7	8.3–9.2	8.8	8.4–9.3	8.7	8.2–9.2	NS	NS	High intake
B. Women	Reference value							General test	Pair-wise comparison	Overall adequacy evaluation^2^
Nutrient	RI	Mean	95% CI	Mean	95% CI	Mean	95% CI	*p*-value	Sign. Diff.^1^	
Education		Low (1)		Middle (2)		High (3)				
Energy (MJ)	-	7.1	6.8–7.4	7.2	6.9–7.5	7.9	7.6–8.1	0,0005	3 > 1, 2	-
Protein (E%)	10–20	17.6	17.1–18.1	17.7	17.2–18.2	17.1	16.5–17.6	NS	NS	High intake
Total Carbohydrates (E%)	45–60	44.8	43.8–45.8	44.9	43.9–45.9	44.5	43.6–45.4	NS	NS	No firm conclusions can be drawn
Fibre (g)	>25	18.5	17.5–19.6	20.8	19.7–22	22.2	21.1–23.3	0.000	3,2 > 1	No firm conclusions can be drawn
Fat (E%)	25–40	37.6	36.7–38.6	37.4	36.5–38.3	38.4	37.6–39.2	NS	NS	Low prevalence of inadequacy
Saturated f.a. (SFA) (E%)	<10	14.6	14–15.2	14.4	13.9–14.9	14.0	13.6–14.4	NS	NS	High intake
Polyunsaturated f.a. (PUFA) (E%)	5–10	6.6	6.3–7	6.7	6.4–7	7.4	7.1–7.7	0.000	3 > 1, 2	Low prevalence of inadequacy
N-3 PUFA (E%)	1	1.6	1.5–1.7	1.6	1.5–1.7	1.8	1.7–1.9	0.0090	3 > 1, 2	Low prevalence of inadequacy
Salt (g)	≤5	6.3	6.1–6.6	6.2	5.9–6.5	6.7	6.4–6.9	NS	NS	High intake
Iron (18–50 years) (mg)	15	9.0	8.5–9.5	10.2	9.6–10.8	10.9	10.3–11.5	0.0000	3, 2 > 1	No firm conclusions can be drawn
Income		Lowest Qrt (1)		Middle (2)		Highest Qrt (3)				
Energy (MJ)	-	7.2	6.8–7.6	7.3	7.1–7.6	7.7	7.4–8.0	NS	NS	-
Protein (E%)	10–20	17.3	16.7–17.9	17.3	17–17.7	17.7	17.1–18.2	NS	NS	High intake
Total Carbohydrates (E%)	45–60	45.8	44.6–46.9	45.1	44.3–45.8	43.5	42.3–44.6	0.021	3 < 1	1,2 low prevalence of inadequacy, 3 no firm conclusions can be drawn
Fibre (g)	>25	20.0	18.7–21.3	20.6	19.5–21.6	21.4	20–22.7	NS	NS	No firm conclusions can be drawn
Fat (E%)	25–40	36.9	35.6–38.2	37.6	36.9–38.3	38.9	37.7–40	NS	1 < 3	Low prevalence of inadequacy
Saturated f.a. (SFA) (E%)	<10	14.0	13.3–14.7	14.5	14.1–14.9	14.6	13.8–15.3	NS	NS	High intake
Polyunsaturated f.a. (PUFA) (E%)	5–10	6.7	6.4–7.1	6.8	6.5–7	7.2	6.9–7.5	0.020	2 < 3	Low prevalence of inadequacy
N-3 PUFA (E%)	1	1.6	1.5–1.7	1.7	1.6–1.8	1.8	1.7–1.9	0.031	1 < 3	Low prevalence of inadequacy
Salt (g)	≤5	6.4	6.1–6.7	6.3	6.1–6.6	6.5	6.2–6.8	NS	NS	High intake
Iron (18–50 years) (mg)	15	9.5	8.9–10.1	10.1	9.5–10.7	10.3	9.6–11.1	NS	NS	No firm conclusions can be drawn
Urbanisation level		Urban (1)		Semi-urban (2)		Rural (3)				
Energy (MJ)	-	7.4	7.2–7.6	7.3	7.0–7.7	7.2	6.9–7.5	NS	NS	-
Protein (E%)	10–20	17.6	17.2–17.9	17.5	16.9–18.1	17.2	16.6–17.7	NS	NS	High intake
Total Carbohydrates (E%)	45–60	44.3	43.6–45	45.6	44.1–47.2	45.2	43.9–46.5	NS	NS	1 no firm conclusions can be drawn, 2,3 low prevalence of inadequacy
Fibre (g)	>25	20.7	19.9–21.6	20.3	18.6–22	19.9	18.2–21.6	NS	NS	No firm conclusions can be drawn
Fat (E%)	25–40	38.2	37.5–38.8	36.9	35.4–38.4	37.6	36.5–38.7	NS	NS	Low prevalence of inadequacy
Saturated f.a. (SFA) (E%)	<10	14.3	13.9–14.6	14.3	13.3–15.3	14.9	14.3–15.4	NS	NS	High intake
Polyunsaturated f.a. (PUFA) (E%)	5–10	7.1	6.8–7.4	6.6	6.3–6.8	6.5	6.2–6.9	0.011	1 > 2, 3	Low prevalence of inadequacy
N-3 PUFA (E%)	1	1.7	1.6–1.8	1.6	1.5–1.7	1.6	1.5–1.7	NS	NS	Low prevalence of inadequacy
Salt (g)	≤5	6.4	6.2–6.6	6.5	6.2–6.8	6.3	5.9–6.6	NS	NS	High intake
Iron (18–50 years) (mg)	15	10.2	9.8–10.6	9.8	8.8–10.8	9.1	8.5–9.8	NS	NS	No firm conclusions can be drawn

^1^ Considered significantly different with group rankings as indicated, if for the general test *p* < 0.05 and for pair-wise comparison *p* < 0.05. ^2^ Using RI reference values provided by Nordic Nutrition Recommendations (NNR2012) [25]. If the mean intake of a group is at or above the RI, there is probably a “low prevalence of inadequacy” and if it is below the RI, “no firm conclusions can be drawn regarding the prevalence of inadequacy at the group level”, according to the NNR2012 [25]. RI, recommended daily intake; E%, % of total energy; f.a., fatty acids; N-3, omega-3.

**Table 4:** Proportion of population groups reaching the average requirement (AR) values, and adequacy evaluation based on usual intake distributions in men and women by education.

	Reference Value	Low (1)		Middle (2)		High (3)		Sign. Diff. ^1^	≥90% of Population Group > AR	Overall Adequacy Evaluation ^2^
Nutrient	AR	%	95% CI	%	95% CI	%	95% CI		Yes/No	
Men										
Vitamin A (µg RE)	600	81	72–90	71	67–77	77	69–85	NS	No	Not adequate
Vitamin D (µg)	7.5	89	85–95	86	79–93	86	80–92	NS	No	Not adequate
Vitamin E (mg)	6	96	94–99	96	95–98	99	97–100	NS	Yes	Adequate
Vitamin B1 (mg)	1.2	65	59–72	65	59–72	64	58–70	NS	No	Not adequate
Vitamin B2 (mg)	1.4	85	80–89	82	78–87	80	76–85	NS	No	Not adequate
Folate (µg)	200	65	59–71	67	61–75	80	73–87	3 > 1	No	Not adequate
Vitamin B12 (µg)	1.4	100	100–100	100	100–100	100	100–100	NS	Yes	Adequate
Vitamin C (mg)	60	70	63–77	72	66–80	82	76–88	NS	No	Not adequate
Calcium (mg)	500	97	95–99	97	95–99	98	96–100	NS	Yes	Adequate
Iron (mg)	7	95	92–99	93	90–97	95	93–98	NS	Yes	Adequate
Iodine (µg)	100	100	99–100	99	98–100	100	99–100	NS	Yes	Adequate
Zinc (mg)	6	99	98–100	99	98–100	99	98–100	NS	Yes	Adequate
Women										
Vitamin A (µg RE)	500	84	74–94	86	80–93	89	82–100	NS	No	Not adequate^3^
Vitamin D (µg)	7.5	69	63–77	70	63–78	69	63–75	NS	No	Not adequate
Vitamin E (mg)	5	97	94–99	96	94–99	100	99–100	3 > 2	Yes	Adequate
Vitamin B1 (mg)	0.9	72	66–77	76	70–83	79	73–86	NS	No	Not adequate
Vitamin B2 (mg)	1.1	90	86–95	91	88–95	91	88–95	NS	Yes	Adequate
Folate (µg)	200	45	38–52	60	55–67	74	68–81	2 > 1, 3 > 1, 2	No	Not adequate
Vitamin B12 (µg)	1.4	100	100–100	100	100–100	100	100–100	NS	Yes	Adequate
Vitamin C (mg)	50	88	82–93	94	91–98	96	94–99	3 > 1	No (1), Yes (2, 3)	1 not adequate, 2,3 adequate
Calcium (mg)	500	96	94–99	98	97–100	98	97–99	NS	Yes	Adequate
Iron (51–74 years) (mg)	6	98	94–100	94	91–98	96	93–100	NS	Yes	Adequate
Iodine (µg)	100	99	97–100	99	98–100	99	98–100	NS	Yes	Adequate
Zinc (mg)	5	100	99–100	100	99–100	99	98–100	NS	Yes	Adequate

^1^ Significant differences in proportions between educational groups were evaluated by non-overlapping 95% CI. ^2^ If the proportion of the group reaching the average requirement (AR) level was ≥90%, the intake was considered “adequate”. If <90% of the group met the AR level, the intake was considered “not adequate”. If over 2.5% of the group exceeded the upper limit of the RI range of macronutrients as E% or exceeded the UL level of micronutrients, the intake was considered “high”. ^3^ Based on the confidence interval, the highest educational group is close to adequate vitamin A intake. RE, retinol equivalents.

**Table 5:** Proportion of population groups reaching the average requirement (AR) values, and adequacy evaluation according to usual intake distributions in men and women by income.

	Reference Value	Lowest Qrt (1)		Middle (2.–3. Qrt) (2)		Highest Qrt (3)		Sign. Diff. ^1^	≥90% of Population Group > AR	Overall Adequacy Evaluation ^2^
Nutrient	AR	%	95% CI	%	95% CI	%	95% CI		Yes/No	
Men										
Vitamin A (µg RE)	600	77	69–88	72	67–80	81	74–90	NS	No	Not adequate
Vitamin D (µg)	7.5	88	80–94	83	77–89	90	84–98	NS	Yes (3), No (1, 2)	3 adequate, 1 and 2 not adequate
Vitamin E (mg)	6	98	96–99	96	94–98	99	99–100	3 > 2	Yes	Adequate
Vitamin B1 (mg)	1.2	63	56–70	63	58–70	67	60–73	NS	No	Not adequate
Vitamin B2 (mg)	1.4	85	80–89	80	75–85	88	83–92	NS	No	Not adequate
Folate (µg)	200	70	62–77	67	62–74	85	76–92	3 > 2	No	Not adequate
Vitamin B12 (µg)	1.4	100	100–100	100	100–100	100	100–100	NS	Yes	Adequate
Vitamin C (mg)	60	72	65–79	70	65–75	93	87–98	3 > 1, 2	Yes (3), No (1, 2)	3 adequate, 1,2 not adequate
Calcium (mg)	500	98	96–99	97	95–99	97	95–99	NS	Yes	Adequate
Iron (mg)	7	94	90–98	95	92–97	97	95–99	NS	Yes	Adequate
Iodine (µg)	100	99	99–100	100	99–100	100	99–100	NS	Yes	Adequate
Zinc (mg)	6	99	98–100	99	98–100	99	99–100	NS	Yes	Adequate
Women										
Vitamin A (µg RE)	500	88	80–100	89	83–96	85	78–94	NS	No	Not adequate
Vitamin D (µg)	7.5	64	58–73	71	64–78	69	61–80	NS	No	Not adequate
Vitamin E (mg)	5	96	94–99	97	95–99	100	99–100	3 > 2	Yes	Adequate
Vitamin B1 (mg)	0.9	72	65–81	76	70–81	78	72–86	NS	No	Not adequate
Vitamin B2 (mg)	1.1	87	82–92	92	88–95	95	93–98	3 > 1	No (1), Yes (2, 3)	1 not adequate, 2,3 adequate
Folate (µg)	200	51	45–60	57	52–62	78	71–87	3 > 1, 2	No	Not adequate
Vitamin B12 (µg)	1.4	100	100–100	100	100–100	100	100–100	NS	Yes	Adequate
Vitamin C (mg)	50	91	86–97	93	89–96	95	93–98	NS	Yes	Adequate
Calcium (mg)	500	95	92–99	98	96–99	99	98–100	NS	Yes	Adequate
Iron (51–74 years) (mg)	6	94	90–99	94	91–97	99	98–100	3 > 2	Yes	Adequate
Iodine (µg)	100	99	97–100	98	96–99	100	99–100	3 > 2	Yes	Adequate
Zinc (mg)	5	99	98–100	100	99–100	100	100–100	NS	Yes	Adequate

^1^ Significant differences in proportions between income groups were evaluated by non-overlapping 95% CI. ^2^ If the proportion of the group reaching the AR level was ≥90%, the intake was considered “adequate”. If <90% of the group met the AR level, the intake was evaluated to be “not adequate”. If over 2.5% of the group exceeded the upper limit of the RI range of macronutrients as E% or exceeded the UL level of micronutrients, the intake was evaluated to be “high”.

**Table 6:** Proportion of population groups reaching the average requirement (AR) values, and adequacy evaluation according to usual intake distributions in men and women by urbanisation level.

	Reference Value	Urban (1)		Semi-Urban (2)		Rural (3)		Sign. Diff. ^1^	≥90% of Population Group > AR	Overall Adequacy Evaluation ^2^
Nutrient	AR	%	95% CI	%	95% CI	%	95% CI		Yes/No	
Men										
Vitamin A (µg RE)	600	74	68–79	83	70–97	72	66–79	NS	No	Not adequate
Vitamin D (µg)	7.5	86	80–91	87	81–93	86	80–93	NS	No	Not adequate
Vitamin E (mg)	6	98	96–99	95	93–98	96	94–100	NS	Yes	Adequate
Vitamin B1 (mg)	1.2	59	53–64	67	61–75	65	60–74	NS	No	Not adequate
Vitamin B2 (mg)	1.4	81	77–86	82	77–87	86	81–95	NS	No	Not adequate
Folate (µg)	200	73	67–78	62	56–69	68	62–77	NS	No	Not adequate
Vitamin B12 (µg)	1.4	100	100–100	100	100–100	100	100–100	NS	Yes	Adequate
Vitamin C (mg)	60	78	72–83	67	59–74	68	63–77	NS	No	Not adequate
Calcium (mg)	500	97	95–98	97	95–99	99	99–100	3 > 1	Yes	Adequate
Iron (mg)	7	94	91–96	97	94–100	92	88–97	NS	Yes	Adequate
Iodine (µg)	100	99	99–100	100	99–100	100	100–100	NS	Yes	Adequate
Zinc (mg)	6	99	98–100	99	98–100	100	99–100	NS	Yes	Adequate
Women										
Vitamin A (µg RE)	500	86	81–92	86	77–96	84	72–96	NS	No	Not adequate
Vitamin D (µg)	7.5	67	61–74	66	61–73	72	64–80	NS	No	Not adequate
Vitamin E (mg)	5	98	96–99	98	96–100	96	93–98	NS	Yes	Adequate
Vitamin B1 (mg)	0.9	75	70–81	71	65–77	76	68–83	NS	No	Not adequate
Vitamin B2 (mg)	1.1	90	87–94	94	90–97	87	84–91	NS	Yes (1, 2), No (3)	1, 2 adequate, 3 not adequate
Folate (µg)	200	65	61–71	56	49–64	44	38–49	3 < 1, 2	No	Not adequate
Vitamin B12 (µg)	1.4	100	100–100	100	99–100	100	100–100	NS	Yes	Adequate
Vitamin C (mg)	50	95	93–98	89	85–94	88	84–93	NS	Yes (1), No (2, 3)	1 adequate, 2, 3 not adequate
Calcium (mg)	500	98	97–99	98	97–99	94	92–97	3 < 1, 2	Yes	Adequate
Iron (51–74 years) (mg)	6	95	92–99	94	90–98	98	96–100	NS	Yes	Adequate
Iodine (µg)	100	99	97–100	99	98–100	99	97–100	NS	Yes	Adequate
Zinc (mg)	5	100	99–100	99	99–100	100	100–100	NS	Yes	Adequate

^1^ Significant differences in proportions between urbanisation level groups were evaluated by non-overlapping 95% CI. ^2^ If the proportion of the group reaching the AR level was ≥90%, the intake was considered “adequate”. If <90% of the group met the AR level, the intake was considered “not adequate”. If over 2.5% of the group exceeded the upper limit of the RI range of macronutrients as E% or exceeded the UL level of micronutrients, the intake was considered “high”.

## Data Availability

The data presented in this study (The FinDiet 2017 Survey data) are available on request from THL Biobank at: https://thl.fi/en/web/thl-biobank/for-researchers (accessed on 14 February 2022). The individual-level data are not publicly available due to privacy restrictions.

## Supplementary Materials

The following supporting information can be downloaded at: https://www.mdpi.com/article/10.3390/nu14071347/s1. Figure S1: Food groups as sources of folate in the diet of men and women according to (a) educational group, (b) income level and (c) urbanisation level (% of daily intake); Table S1: Proportions of population groups reaching the recommended daily intake (RI) values according to usual intake distributions in men and women by education, income and urbanisation level; Table S2: Proportions of population groups exceeding the upper value of macronutrient RI range (E%) or the UL value [26], and evaluation of the intakes; Table S3: Average nutrient intakes in men and women by education, income and urbanisation level; Table S4: Nutrient intakes from food and combined intakes from food and food supplements, and proportion of men and women reaching the dietary reference intakes, modified from [28].
