# Supplementary material for: Disparities in Nutritional Adequacy of Diets between Different Socioeconomic Groups of Finnish Adults
Source: Nutrients. 2022 Mar 23;14(7):1347. doi: 10.3390/nu14071347 (PMC9002951; doi:10.3390/nu14071347)
Supplement: Supplementary file 1 [file nutrients-14-01347-s001.zip › nutrients-1621256-supplementary.pdf]

Table S1. Proportions of population groups reaching the recommended daily intake (RI) values according to usual intake distributions in men and women by education, income and urbanisation level.

		**Education**							**Income**							**Urbanisation level**						
	**Reference value**	**Low (1)**		**Middle (2)**		**High (3)**		**Sign. Diff.[2]**	**Lowest Qrt (1)**		**Middle (2.-3. Qrt) (2)**		**Highest Qrt (3)**		**Sign. Diff.[2]**	**Urban (1)**		**Semi-urban (2)**		**Rural (3)**		**Sign. Diff.[2]**
**Nutrient**	**RI[1]**	**%**	**95% CI**	**%**	**95% CI**	**%**	**95% CI**		**%**	**95% CI**	**%**	**95% CI**	**%**	**95% CI**		**%**	**95% CI**	**%**	**95% CI**	**%**	**95% CI**	
**Men**																						
Protein (E%)	10-20	100	100–100	100	100–100	100	100–100	NS	100	100–100	100	100–100	100	100–100	NS	100	100–100	100	100–100	100	100–100	NS
Total carbohydrates (E%)	45-60	45	40–52	31	23–38	41	35–47	1>2	32	23–40	45	41–51	37	30–43	2>1	39	34–45	33	26–41	53	47–62	3>1,2
Fibre (g)	> 35	4	1–7	4	2–6	12	7–16	3>1,2	5	2–8	6	3–8	7	3–10	NS	5	3–7	5	2–8	6	1–10	NS
Fat (E%)	25-40	100	99–100	100	100–100	99	99–100	NS	100	99–100	100	100–100	100	99–100	NS	100	100–100	100	100–100	100	99–100	NS
Saturated f.a. (SFA) (E%)	<10	1	0–3	1	0–2	7	4–11	3>1,2	4	2–7	1	0–3	3	1–5	NS	3	1–5	2	1–4	2	0–5	NS
Polyunsaturated f.a. (PUFA) (E%)	5-10	97	92–100	94	89–99	97	93–100	NS	98	94–100	92	88–97	97	94–100	NS	97	93–99	97	93–100	82	75–91	3<1,2
N-3 PUFA (E%)	1	98	94–100	98	94–100	96	92–99	NS	99	95–100	94	89–98	99	97–100	NS	98	96–100	97	91–100	85	78–96	3<1
Salt (g)	≤5	3	1–4	2	0–3	3	1–5	NS	2	0–3	3	1–5	2	1–4	NS	3	2–5	2	0–3	2	0–4	NS
**Women**																						
Protein (E%)	10-20	100	100–100	100	100–100	100	100–100	NS	100	100–100	100	100–100	100	100–100	NS	100	100–100	100	100–100	100	100–100	NS
Total carbo hydrates (E%)	45-60	50	43–56	51	45–56	53	41–64	NS	57	50–64	50	44–55	41	36–48	1>3	46	40–52	56	50–65	52	47–57	NS
Fibre (g)	>25	14	10–19	24	19–29	32	26–36	2,3>1	20	16–26	21	17–25	26	22–31	NS	23	19–28	23	18–29	19	13–24	NS
Fat (E%)	25-40	100	99–100	99	98–100	100	99–100	NS	99	98–100	100	99–100	100	99–100	NS	100	99–100	97	95–99	99	98–100	2<1
Saturated f.a. (SFA) (E%)	<10	6	3–9	6	1–10	6	2–10	NS	6	2–11	5	2–8	4	1–7	NS	5	2–8	14	9–17	4	1–6	**2>1,3**
Polyunsaturated f.a. (PUFA) (E%)	5-10	85	79–91	90	85–96	94	90–98	NS	89	83–96	90	85–94	95	91–100	NS	91	87–94	96	90–100	86	80–92	NS
N-3 PUFA (E%)	1	88	82–94	92	88–96	98	95–100	3>1	93	87–99	92	88–96	97	93–100	NS	94	91–97	93	87–99	93	87–100	NS
Salt (g)	≤5	15	8–21	16	10–20	11	5–15	NS	15	9–20	14	9–19	15	9–20	NS	14	9–18	15	9–20	18	12–24	NS
Iron (18-50 years) (mg)	15	1	0–2	3	1–6	7	2–10	3>1	1	0–3	3	1–5	3	0–5	NS	4	1–6	3	1–6	1	0–2	NS

[1] For protein, total carbohydrates, fat and polyunsaturated fatty acids the lower bound of RI is used for the analyses. [2] Significant differences in proportions between socio-demographic groups were evaluated by non-overlapping 95% CI.

Table S2. Proportions of population groups exceeding the upper value of macronutrient RI range (E%) or the UL value [26] and evaluation of the intakes.

		Education					Income					Urbanisation level					
Nutrient		Low (1)	Middle (2)	High (3)		Evaluation	Lowest Qrt (1)	Middle (2)	Highest Qrt (3)		Evaluation	Urban (1)	Semi-urban (2)	Rural (3)		Evaluation	Overall evaluation[1]
**Men**	**Upper value of the RI range (E%) or the UL**	**%**	**%**	**%**	**Sign. Diff.[2]**	**< 2.5% of population above the upper value of the RI range or > UL**	**%**	**%**	**%**	**Sign. Diff.[2]**	**< 2.5% of population above the upper value of the RI range or > UL**	**%**	**%**	**%**	**Sign. Diff.[2]**	**< 2.5% of population above the upper value of the RI range or > UL**	
Protein (E%)	20	18.1	21.9	25.2	NS	No	29.8	16.2	32.9	NS	No	27.1	16.9	4.8	NS	No	High intake
Total carbohydrates (E%)	60	0.4	0.0	0.2	NS	Yes	0.0	0.1	0.2	NS	Yes	0.1	0.0	0.1	NS	Yes	Safe intake
Fat (E%)	40	30.2	41.5	39.3	NS	No	41.8	30.8	37.6	NS	No	36.5	37.3	30.2	NS	No	High intake
Saturated f.a. (SFA) (E%)	10	98.6	98.9	92.9	3<1,2	No	95.9	98.7	97.0	NS	No	96.9	97.5	98.1	NS	No	High intake
Polyunsaturated f.a. (PUFA) (E%)	10	0.0	0.8	1.1	NS	Yes	0.4	1.0	0.3	NS	Yes	0.9	0.1	0.2	NS	Yes	Safe intake
Salt (g)	5	97.5	98.3	97.0	NS	No	98.4	97.2	97.6	NS	No	96.7	98.5	97.7	NS	No	High intake
Vitamin D (µg)	100	0.0	0.0	0.0	NS	Yes	0.0	0.0	0.0	NS	Yes	0.0	0.0	0.0	NS	Yes	Safe intake
Vitamin E (mg)	300	0.0	0.0	0.0	NS	Yes	0.0	0.0	0.0	NS	Yes	0.0	0.0	0.0	NS	Yes	Safe intake
Folate (µg)	1000	0.0	0.0	0.0	NS	Yes	0.0	0.0	0.0	NS	Yes	0.0	0.0	0.0	NS	Yes	Safe intake
Vitamin C (mg)	1000	0.0	0.0	0.0	NS	Yes	0.0	0.0	0.0	NS	Yes	0.0	0.0	0.0	NS	Yes	Safe intake
Calcium (mg)	2500	0.6	1.1	0.6	NS	Yes	1.1	0.6	1.5	NS	Yes	0.7	0.8	0.1	NS	Yes	Safe intake
Iron (mg)	25	0.0	0.2	0.3	NS	Yes	0.3	0.1	0.1	NS	Yes	0.2	0.0	0.3	NS	Yes	Safe intake
Iodine (µg)	600	0.0	0.0	0.0	NS	Yes	0.0	0.0	0.0	NS	Yes	0.0	0.0	0.0	NS	Yes	Safe intake
Zinc (mg)	25	0.2	0.6	0.1	NS	Yes	0.8	0.2	0.2	NS	Yes	0.4	0.1	0.1	NS	Yes	Safe intake
**Women**																	
Protein (E%)	20	18.9	19.4	4.0	NS	No	11.0	16.0	21.1	NS	No	16.0	14.6	8.6	NS	No	High intake
Total carbohydrates (E%)	60	0.4	0.3	0.0	NS	Yes	0.1	0.2	0.1	NS	Yes	0.0	1.2	0.3	1<2,3	Yes	Safe intake
Fat (E%)	40	28.3	28.7	31.0	NS	No	25.8	30.5	35.4	NS	No	31.6	24.4	31.2	NS	No	High intake
Saturated f.a. (SFA) (E%)	10	94.1	94.2	93.5	NS	No	93.6	94.9	95.7	NS	No	95.4	86.0	96.4	2>1,3	No	High intake
Polyunsaturated f.a. (PUFA) (E%)	10	1.5	1.3	4.8	NS	1,2 Yes, 3 No	2.9	1.5	2.2	NS	Yes	4.2	0.0	1.4	1>2	1 No, 2,3 Yes	Safe intake, but high intake edu3, income 1, and urban women
Salt (g)	5	85.0	84.0	89.0	NS	No	85.0	85.8	84.9	NS	No	86.0	85.0	82.0	NS	No	High intake
Vitamin D (µg)	100	0.0	0.0	0.0	NS	Yes	0.0	0.0	0.0	NS	Yes	0.0	0.0	0.0	NS	Yes	Safe intake
Vitamin E (mg)	300	0.0	0.0	0.0	NS	Yes	0.0	0.0	0.0	NS	Yes	0.0	0.0	0.0	NS	Yes	Safe intake
Folate (µg)	1000	0.0	0.0	0.0	NS	Yes	0.0	0.0	0.0	NS	Yes	0.0	0.0	0.0	NS	Yes	Safe intake
Vitamin C (mg)	1000	0.0	0.0	0.0	NS	Yes	0.0	0.0	0.0	NS	Yes	0.0	0.0	0.0	NS	Yes	Safe intake
Calcium (mg)	2500	0.0	0.0	0.0	NS	Yes	0.0	0.0	0.0	NS	Yes	0.0	0.0	0.1	NS	Yes	Safe intake
Iron (mg)	25	0.0	0.0	0.0	NS	Yes	0.0	0.0	0.0	NS	Yes	0.0	0.0	0.0	NS	Yes	Safe intake

Iodine (µg)	600	0.0	0.0	0.0	NS	Yes	0.0	0.0	0.0	NS	Yes	0.0	0.0	0.0	NS	Yes	Safe intake
Zinc (mg)	25	0.0	0.0	0.0	NS	Yes	0.0	0.0	0.0	NS	Yes	0.0	0.0	0.0	NS	Yes	Safe intake

[1] If >2.5% of the population group exceeded the upper limit of the recommended daily intake (RI) range or the upper intake level (UL), the intake was evaluated to be "high". If below, the intake was considered "safe". [2] Significant differences in proportions between socio-demographic groups were evaluated by non-overlapping 95% CI.

Table S3. Average nutrient intakes in men and women by education, income and urbanisation level.

**Men**								
**Education**	Low (1)		Middle (2)		High (3)		General test	Pair-wise comparison
**Nutrient**	Mean	95% CI	Mean	95% CI	Mean	95% CI	P-value	Sign. Diff.[1]
Vitamin A (µg RE)	953	840–1067	850	741–960	909	769–1049	NS	NS
Vitamin D (µg)	13.7	12.5–15	12.3	10.9–13.8	12.4	11.4–13.5	NS	NS
Vitamin E (mg)	11.5	10.9–12.1	11.7	10.7–12.7	12.4	11.7–13.1	0.004	3>1,2
Vitamin B1 (mg)	1.4	1.3–1.4	1.3	1.3–1.4	1.3	1.3–1.4	NS	NS
Vitamin B2 (mg)	2.0	1.9–2.1	1.9	1.8–2.1	1.9	1.8–2	NS	NS
Folate (µg)	236	224–247	238	222–254	261	247–275	0.000	3>1,2
Vitamin B12 (µg)	6.8	6.2–7.5	6.3	5.6–6.9	6.5	5.9–7.1	NS	NS
Vitamin C (mg)	92	82–102	90	80–101	114	104–125	0.001	3>1,2
Calcium (mg)	1182	1100–1263	1186	1084–1287	1174	1109–1240	NS	NS
Iron (mg)	11.2	10.5–11.8	11.4	10.8–12.1	12.0	11.4–12.5	0.007	3>1,2
Iodine (µg)	235	224–245	238	221–256	236	224–247	NS	NS
Zinc (mg)	12.8	12.2–13.5	13.2	12.3–14.1	12.4	11.8–12.9	NS	NS
Income	Lowest Qrt (1)		Middle (2.-3. Qrt) (2)		Highest Qrt (3)		General test	Pair-wise comparison
Nutrient	Mean	95% CI	Mean	95% CI	Mean	95% CI	P-value	Sign. Diff.[1]
Vitamin A (µg RE)	960	837–1084	845	753–937	977	803–1152	NS	NS
Vitamin D (µg)	13.4	11.9–15	12.8	11.8–13.8	12.1	10.9–13.4	NS	NS
Vitamin E (mg)	12.0	10.9–13.1	11.6	11.1–12.2	12.3	11.5–13	NS	NS
Vitamin B1 (mg)	1.3	1.3–1.4	1.4	1.3–1.4	1.4	1.3–1.4	NS	NS
Vitamin B2 (mg)	2.1	2–2.2	1.9	1.8–2	2.0	1.9–2.1	NS	NS
Folate (µg)	249	232–266	238	226–250	257	242–272	NS	NS
Vitamin B12 (µg)	6.7	6–7.5	6.0	5.6–6.5	7.3	6.5–8.1	0.019	2<3
Vitamin C (mg)	92	79–105	95	86–105	115	103–127	0.008	1<3, 2<3
Calcium (mg)	1260	1149–1370	1138	1079–1197	1192	1082–1303	NS	NS
Iron (mg)	11.5	10.7–12.3	11.2	10.7–11.7	12.2	11.5–12.9	0.014	1<3
Iodine (µg)	245	226–263	231	221–240	237	221–253	NS	NS
Zinc (mg)	13.4	12.5–14.4	12.4	11.9–12.9	13.2	12.3–14	NS	NS
Urbanisation level	Urban (1)		Semi-urban (2)		Rural (3)		General test	Pair-wise comparison
Nutrient	Mean	95% CI	Mean	95% CI	Mean	95% CI	P-value	Sign. Diff.[1]
Vitamin A (µg RE)	893	824–963	919	782–1055	924	682–1167	NS	NS
Vitamin D (µg)	12.8	11.8–13.8	12.4	11.2–13.7	13.9	11.9–15.9	NS	NS
Vitamin E (mg)	12.1	11.4–12.8	11.7	11–12.4	10.9	9.8–11.9	NS	NS
Vitamin B1 (mg)	1.3	1.3–1.4	1.4	1.3–1.5	1.4	1.3–1.5	NS	NS
Vitamin B2 (mg)	1.9	1.8–2	2.0	1.9–2.1	2.0	1.9–2.1	NS	NS
Folate (µg)	250	238–262	233	218–247	240	221–259	NS	NS
Vitamin B12 (µg)	6.5	6–6.9	6.5	5.8–7.1	6.8	5.5–8.2	NS	NS
Vitamin C (mg)	104	95–112	87	78–95	93	80–107	0.026	1>2
Calcium (mg)	1169	1094–1244	1192	1099–1285	1207	1106–1309	0.026	1<3
Iron (mg)	11.6	11.1–12.1	11.2	10.6–11.9	11.5	10.2–12.8	NS	NS
Iodine (µg)	236	223–248	236	226–247	235	221–250	NS	NS
Zinc (mg)	12.8	12.2–13.4	13.0	12.2–13.8	12.7	11.9–13.5	NS	NS

**Women**								
Education	Low (1)		Middle (2)		High (3)		General test	Pair-wise comparison
Nutrient	Mean	95% CI	Mean	95% CI	Mean	95% CI	P-value	Sign. Diff.[1]
Vitamin A (μg RE)	666	609–723	764	681–848	824	729–918	0,003	3,2>1
Vitamin D (μg)	9.6	8.9–10.4	9.3	8.8–9.8	10.0	9.3–10.7	NS	NS
Vitamin E (mg)	9.5	8.9–10.1	10.0	9.4–10.7	11.5	10.9–12	0,000	3>1,2
Vitamin B1 (mg)	1.0	1–1.1	1.1	1–1.1	1.1	1–1.1	NS	NS
Vitamin B2 (mg)	1.6	1.5–1.6	1.6	1.5–1.7	1.6	1.6–1.7	NS	NS
Folate (μg)	198	186–210	221	211–231	241	228–254	0,000	3,2>1
Vitamin B12 (μg)	4.7	4.3–5.1	4.8	4.4–5.2	5.3	4.8–5.7	NS	NS
Vitamin C (mg)	98	87–109	114	102–125	120	110–130	0,001	3,2>1
Calcium (mg)	961	911–1010	978	933–1024	1025	980–1069	NS	NS
Iron (51-74 years) (mg)	9.43	8.8–10.1	9.14	8.6–9.6	9.93	9.2–10.6	NS	NS
Iodine (μg)	183	174–191	183	175–191	194	186–202	NS	NS
Zinc (mg)	9.4	9–9.7	9.5	9.1–10	10.0	9.6–10.4	NS	NS
Income	Lowest Qrt (1)		Middle (2.-3. Qrt) (2)		Highest Qrt (3)		General test	Pair-wise comparison
Nutrient	Mean	95% CI	Mean	95% CI	Mean	95% CI	P-value	Sign. Diff.[1]
Vitamin A (μg RE)	675	609–742	786	706–865	764	697–830	NS	NS
Vitamin D (μg)	8.9	8.1–9.8	9.9	9.3–10.4	10.0	9.3–10.7	0.039	1<3
Vitamin E (mg)	9.7	8.9–10.4	10.0	9.5–10.5	11.5	10.8–12.2	0.000	3>1,2
Vitamin B1 (mg)	1.0	1–1.1	1.1	1–1.1	1.1	1–1.1	NS	NS
Vitamin B2 (mg)	1.5	1.4–1.6	1.6	1.6–1.7	1.7	1.6–1.8	0.027	1<3
Folate (μg)	206	194–217	223	212–233	235	224–245	0.000	3>1,2
Vitamin B12 (μg)	4.5	4.1–4.8	4.9	4.6–5.3	5.3	4.9–5.8	0.021	1<3
Vitamin C (mg)	107	91–123	109	101–118	121	111–131	0.003	1<3
Calcium (mg)	937	872–1001	986	955–1017	1057	1003–1112	0.004	NS
Iron (51-74 years) (mg)	9.02	8.3–9.7	9.56	9.1–10	9.87	9.3–10.4	NS	NS
Iodine (μg)	181	173–190	186	179–193	194	185–203	NS	NS
Zinc (mg)	9.3	8.8–9.9	9.6	9.3–9.8	10.1	9.6–10.6	NS	NS
Urbanisation level	Urban (1)		Semi-urban (2)		Rural (3)		General test	Pair-wise comparison
Nutrient	Mean	95% CI	Mean	95% CI	Mean	95% CI	P-value	Sign. Diff.[1]
Vitamin A (μg RE)	767	706–828	728	615–841	703	637–769	NS	NS
Vitamin D (μg)	9.4	9–9.9	9.9	9.2–10.6	10.0	9–11	NS	NS
Vitamin E (mg)	10.6	10.2–11.1	9.8	9.1–10.6	9.5	8.8–10.2	NS	NS
Vitamin B1 (mg)	1.1	1–1.1	1.1	1–1.1	1.1	1–1.1	NS	NS
Vitamin B2 (mg)	1.6	1.5–1.6	1.7	1.6–1.7	1.6	1.5–1.7	NS	NS
Folate (μg)	227	218–236	213	198–227	199	184–213	0.016	1>3
Vitamin B12 (μg)	4.9	4.6–5.2	5.0	4.4–5.6	4.7	4.2–5.2	NS	NS
Vitamin C (mg)	119	110–128	99	88–110	98	85–111	0.048	NS
Calcium (mg)	985	951–1020	1005	950–1060	962	899–1025	NS	NS
Iron (51-74 years) (mg)	9.52	9.1–10	9.19	8.6–9.8	9.55	8.8–10.3	NS	NS
Iodine (μg)	183	178–189	192	183–201	189	175–203	NS	NS
Zinc (mg)	9.6	9.3–9.9	9.6	9.1–10	9.8	9.4–10.2	NS	NS

[1] Considered significantly different and marked with numbers, if the general test $p < 0.05$ and pair-wise comparison $p<0.05$.

Table S4. Nutrient intakes from food and combined intakes from food and food supplements and proportion of men and women reaching the dietary reference intakes, modified from [28].

						**Evaluation**						
Nutrient	Source	AR/RI	AR	>AR%	>90% >AR	RI	Mean	Mean according to the RI	Upper value of the RI range (E%) or the UL	% of population >the upper value of the RI range or >UL	<2.5% of population above the upper value of the RI range or >UL	Overall evaluation
**Men**												
Fat (E%)	Food	RI	NA[1]	NA[1]	NA[1]	25-40	38.7	Yes	40	n.a.[2]	n.a.[2]	n.a.[2]
Saturated f.a. (E%)	Food	RI	NA[1]	NA[1]	NA[1]	<10	15.1	No	10	n.a.[2]	n.a.[2]	n.a.[2]
Monounsaturated f.a. (E%)	Food	RI	NA[1]	NA[1]	NA[1]	10-20	14.6	Yes	20	n.a.[2]	n.a.[2]	n.a.[2]
Polyunsaturated f.a. (E%)	Food	RI	NA[1]	NA[1]	NA[1]	5-10	6.8	Yes	10	n.a.[2]	n.a.[2]	n.a.[2]
N-3 polyunsaturated f.a. (E%)	Food	RI	NA[1]	NA[1]	NA[1]	1	1.6	Yes	NA[1]	NA[1]	NA[1]	NA[1]
Carbohydrates (E%)	Food	RI	NA[1]	NA[1]	NA[1]	45-60	41.3	No	60	n.a.[2]	n.a.[2]	n.a.[2]
Protein (E%)	Food	RI	NA[1]	NA[1]	NA[1]	10-20	18	Yes	20	n.a.[2]	n.a.[2]	n.a.[2]
Vitamin A (µg RE)	Food	AR	600	76	No	NA[1]	NA[1]	NA[1]	n.a.[2]	NA[1]	NA[1]	NA[1]
	Food and supplements	AR	600	82	No	NA[1]	NA[1]	NA[1]	n.a.[2]	NA[1]	NA[1]	NA[1]
Vitamin D (µg)	Food	AR	7.5	86	No	NA[1]	NA[1]	NA[1]	100	0	Yes	Safe intake
	Food and supplements	AR	7.5	93	Yes	NA[1]	NA[1]	NA[1]	100	0.9	Yes	Safe intake
Vitamin E (mg)	Food	AR	6	97.2	Yes	NA[1]	NA[1]	NA[1]	300	0	Yes	Safe intake
	Food and supplements	AR	6	97.7	Yes	NA[1]	NA[1]	NA[1]	300	0	Yes	Safe intake
Vitamin B1 (Thiamine) (mg)	Food	AR	1.2	64	No	NA[1]	NA[1]	NA[1]	n.a.[2]	NA[1]	NA[1]	NA[1]
	Food and supplements	AR	1.2	77	No	NA[1]	NA[1]	NA[1]	n.a.[2]	NA[1]	NA[1]	NA[1]
Vitamin B2 (Riboflavin) (mg)	Food	AR	1.4	82	No	NA[1]	NA[1]	NA[1]	n.a.[2]	NA[1]	NA[1]	NA[1]
	Food and supplements	AR	1.4	89	No	NA[1]	NA[1]	NA[1]	n.a.[2]	NA[1]	NA[1]	NA[1]
Folate (µg)	Food	AR	200	71	No	NA[1]	NA[1]	NA[1]	1000	0	Yes	Safe intake
	Food and supplements	AR	200	80	No	NA[1]	NA[1]	NA[1]	1000	0.03	Yes	Safe intake
Vitamin B12 (µg)	Food	AR	1.4	99.9	Yes	NA[1]	NA[1]	NA[1]	n.a.[2]	NA[1]	NA[1]	NA[1]
	Food and supplements	AR	1.4	100	Yes	NA[1]	NA[1]	NA[1]	n.a.[2]	NA[1]	NA[1]	NA[1]
Vitamin C (mg)	Food	AR	60	75	No	NA[1]	NA[1]	NA[1]	1000	0	Yes	Safe intake
	Food and supplements	AR	60	84	No	NA[1]	NA[1]	NA[1]	1000	0.9	Yes	Safe intake
Salt (g)	Food	RI	NA[1]	NA[1]	NA[1]	5	2.4	No	n.a.[2]	NA[1]	NA[1]	NA[1]
	Food and supplements	RI	NA[1]	NA[1]	NA[1]	NA[1]	NA[1]	No	n.a.[2]	NA[1]	NA[1]	NA[1]
Calcium (mg)	Food	AR	500	97.3	Yes	NA[1]	NA[1]	NA[1]	2500	0.6	Yes	Safe intake
	Food and supplements	AR	500	97.7	Yes	NA[1]	NA[1]	NA[1]	2500	0.6	Yes	Safe intake
Iron (mg)	Food	AR	7	94.8	Yes	NA[1]	NA[1]	NA[1]	25	0.09	Yes	Safe intake
	Food and supplements	AR	7	95.3	Yes	NA[1]	NA[1]	NA[1]	25	0.1	Yes	Safe intake
Iodine (µg)	Food	AR	100	99.6	Yes	NA[1]	NA[1]	NA[1]	600	0	Yes	Safe intake
	Food and supplements	AR	100	99.7	Yes	NA[1]	NA[1]	NA[1]	600	0	Yes	Safe intake
Zinc (mg)	Food	AR	6	99.2	Yes	NA[1]	NA[1]	NA[1]	25	0.2	Yes	Safe intake
	Food and supplements	AR	6	99.5	Yes	NA[1]	NA[1]	NA[1]	25	4	No	High intake

**Women**												
Fat (E%)	Food	RI	NA[1]	NA[1]	NA[1]	25-40	37.7	Yes	40	n.a.[2]	n.a.[2]	n.a.[2]
Saturated f.a. (E%)	Food	RI	NA[1]	NA[1]	NA[1]	<10	14.4	No	10	n.a.[2]	n.a.[2]	n.a.[2]
Monounsaturated f.a. (E%)	Food	RI	NA[1]	NA[1]	NA[1]	10-20	14.3	Yes	20	n.a.[2]	n.a.[2]	n.a.[2]
Polyunsaturated f.a. (E%)	Food	RI	NA[1]	NA[1]	NA[1]	5-10	6.9	Yes	10	n.a.[2]	n.a.[2]	n.a.[2]
N-3 polyunsaturated f.a. (E%)	Food	RI	NA[1]	NA[1]	NA[1]	1	1.7	Yes		NA[1]	NA[1]	NA[1]
Carbohydrates (E%)	Food	RI	NA[1]	NA[1]	NA[1]	45-60	42.5	No	60	n.a.[2]	n.a.[2]	n.a.[2]
Protein (E%)	Food	RI	NA[1]	NA[1]	NA[1]	10-20	17.5	Yes	20	n.a.[2]	n.a.[2]	n.a.[2]
Vitamin A (µg RE)	Food	AR	500	14	No	NA[1]	NA[1]	NA[1]	n.a.[2]	NA[1]	NA[1]	NA[1]
	Food and supplements	AR	500	10	No	NA[1]	NA[1]	NA[1]	n.a.[2]	NA[1]	NA[1]	NA[1]
Vitamin D (µg)	Food	AR	7.5	30	No	NA[1]	NA[1]	NA[1]	100	0	Yes	Safe intake
	Food and supplements	AR	7.5	7	Yes	NA[1]	NA[1]	NA[1]	100	1.3	Yes	Safe intake
Vitamin E (mg)	Food	AR	5	2.4	Yes	NA[1]	NA[1]	NA[1]	300	0	Yes	Safe intake
	Food and supplements	AR	5	1.3	Yes	NA[1]	NA[1]	NA[1]	300	0	Yes	Safe intake
Vitamin B1 (Thiamine) (mg)	Food	AR	0.9	25	No	NA[1]	NA[1]	NA[1]	n.a.[2]	NA[1]	NA[1]	NA[1]
	Food and supplements	AR	0.9	12	No	NA[1]	NA[1]	NA[1]	n.a.[2]	NA[1]	NA[1]	NA[1]
Vitamin B2 (Riboflavin) (mg)	Food	AR	1.1	9	Yes	NA[1]	NA[1]	NA[1]	n.a.[2]	NA[1]	NA[1]	NA[1]
	Food and supplements	AR	1.1	5	Yes	NA[1]	NA[1]	NA[1]	n.a.[2]	NA[1]	NA[1]	NA[1]
Folate (µg)	Food	AR	200	38	No	NA[1]	NA[1]	NA[1]	1000	0	Yes	Safe intake
	Food and supplements	AR	200	21	No	NA[1]	NA[1]	NA[1]	1000	0.2	Yes	Safe intake
Vitamin B12 (µg)	Food	AR	1.4	0.05	Yes	NA[1]	NA[1]	NA[1]	n.a.[2]	NA[1]	NA[1]	NA[1]
	Food and supplements	AR	1.4	0.01	Yes	NA[1]	NA[1]	NA[1]	n.a.[2]	NA[1]	NA[1]	NA[1]
Vitamin C (mg)	Food	AR	50	7	Yes	NA[1]	NA[1]	NA[1]	1000	0	Yes	Safe intake
	Food and supplements	AR	50	4	Yes	NA[1]	NA[1]	NA[1]	1000	0.4	Yes	Safe intake
Salt (g)	Food	RI	NA[1]	NA[1]	NA[1]	5	14	No	n.a.[2]	NA[1]	NA[1]	NA[1]
	Food and supplements	RI	NA[1]	NA[1]	NA[1]	NA[1]	NA[1]	No	n.a.[2]	NA[1]	NA[1]	NA[1]
Calcium (mg)	Food	AR	500	2.5	Yes	NA[1]	NA[1]	NA[1]	2500	0	Yes	Safe intake
	Food and supplements	AR	500	1.8	Yes	NA[1]	NA[1]	NA[1]	2500	0.07	Yes	Safe intake
Iron (mg)	Food	AR	10	NA	NA1	15	NA[1]	NA[1]	25	0	Yes	Safe intake
Pre-menopausal women	Food and supplements	AR	10	NA	NA1	15	n.a.[2]	n.a.[2]	25	0.01	Yes	Safe intake
Iron (mg)	Food	AR	6	n.a.[2]	n.a.[2]	9	n.a.[2]	n.a.[2]	25	0	Yes	Safe intake
Post-menopausal women	Food and supplements	AR	6	n.a.[2]	n.a.[2]	9	n.a.[2]	n.a.[2]	25	0.01	Yes	Safe intake
Iodine (µg)	Food	AR	100	1.3	Yes	NA[1]	NA[1]	NA[1]	600	0	Yes	Safe intake
	Food and supplements	AR	100	1.1	Yes	NA[1]	NA[1]	NA[1]	600	0	Yes	Safe intake
Zinc (mg)	Food	AR	0.4	7	Yes	NA[1]	NA[1]	NA[1]	25	0	Yes	Safe intake
	Food and supplements	AR	0.4	7	Yes	NA[1]	NA[1]	NA[1]	25	2.7	No	High intake

[1]Assessment not applicable [2] Data or value not available RI, recommended daily intake; AR, average requirement; UL, upper intake level.

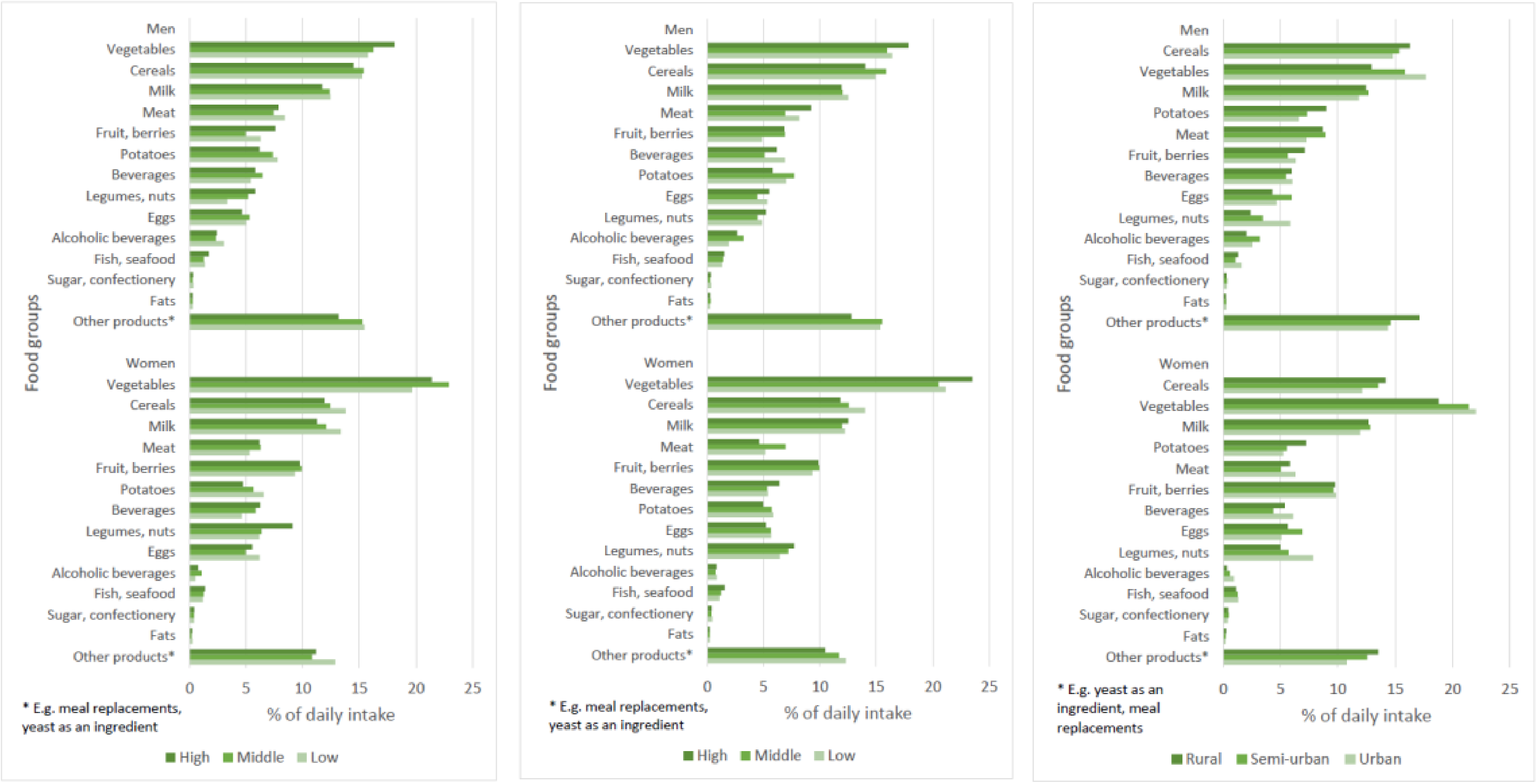

(a) (b) (c)

Figure S1. Food groups as sources of folate in the diet of men and women according to the (a) educational group, (b) income level and (c) urbanisation level (% of daily intake).
